# Italian and Middle Eastern adherence to Mediterranean diet in relation to Body Mass Index and non-communicable diseases: nutritional adequacy of simulated weekly food plans

**DOI:** 10.1186/s12967-024-05325-1

**Published:** 2024-07-30

**Authors:** Ester Luconi, Martina Tosi, Patrizia Boracchi, Ilaria Colonna, Emilia Rappocciolo, Anita Ferraretto, Erna C. Lorenzini

**Affiliations:** 1 Department of Biomedical Sciences for Health, via Luigi Mangiagalli 31, Milano, 20133 Italy; 2 Department of Health Sciences, University of Milan, Via di Rudinì 8, Milano, 20146 Italy; 3 Department of Biomedical and Clinical Sciences, University of Milan, Via Giovanni Battista Grassi 74, Milano, 20157 Italy; 4 University of Milan, Milano, Italy; 5 Biology and Biochemistry Department, Birzeit University, PO BOX 14, Birzeit, Palestine

**Keywords:** Non-communicable diseases, Food-based dietary guidelines, Mediterranean diet adherence, Italian dietary food plan, Middle eastern dietary food plan, Macro and micronutrients, Meta-analysis, Body mass index, Medical statistics.

## Abstract

**Background:**

The Mediterranean diet (MD), known to prevent obesity, overweight and the related non communicable diseases (NCD), is based on typical dishes, foods and on a common cultural milieu. Although MD is the basis of dietary guidelines, the prevalence of obesity, overweight and NCD, is increasing both in Western regions, and even more in Middle Eastern regions (MER). This study aimed to analyze (i) the impact of different levels of adherence to the MD, in Italy and MER, on body mass index (BMI) (ii) the bromatological composition of a simulated 7-days food plan (7-DFP) based on Italian or MER typical meals, following MD criteria and the Italian or MER food base dietary guideline; (iii) the optimization of nutrients impacting on NCD.

**Methods:**

The 7-DFPs were implemented using a dietary software. The association between adherence to MD and BMI was evaluated by pooled estimated ORs (with 95% confidence intervals and p-values). Pooled measures were obtained by the methods appropriate for meta-analysis. The different food-based guidelines have been compared.

**Results:**

The pooled ORs of obese status comparing medium vs. high adherence to MD were: 1.19 (95% C.I.: 0.99; 1.42, p-value = 0.062) and 1.12 (95% C.I.: 0.90; 1.38, p-value = 0.311) for MER and Italy respectively. For the comparison of low vs. high adherence, the pooled ORs were 1.05 (95% C.I.: 0.88; 1.24, p-value = 0.598) for MER, and 1.20 (95% C.I.: 1.02; 1.41, p-value = 0.031) for Italy when outliers are removed. High adherence to the MD resulted as potential protective factor against obesity. In MER 7-DFP: total fats is higher (34.5 E%) vs. Italian 7-DFP (29.4 E%); EPA (20 mg) and DHA (40 mg) are lower than recommended (200 mg each); sugars (12.6 E%) are higher than recommended (< 10 E%). Calcium, Zinc, and vitamin D do not reach target values in both 7-DFPs.

**Conclusion:**

This study highlights that, even when 7-DFPs follow MD and refer to nutrient needs, it is necessary to verify nutrient excesses or deficits impacting on NCD. High MD adherence is protective toward NCDs. MD principles, and energy balance should be communicated according to socioeconomic and educational levels.

**Supplementary Information:**

The online version contains supplementary material available at 10.1186/s12967-024-05325-1.

## Background

Non-communicable diseases (NCD), such as diabetes, hypertension, stroke, and cardiovascular diseases (CVD), are often linked to metabolic imbalance and are among the leading causes of death and disability in both developed and developing countries [1–3]. From a metabolic perspective, NCD are mainly linked to obesity and overweight; obesity is, in fact, the sixth leading cause of global disease burden [4, 5] and, along with overweight, significantly increases the risk of NCD. Obesity and overweight are mainly due to unbalanced diets and sedentary lifestyles, which are modifiable self-managed risk factors and are associated with NCD [6]. Overnutrition causes, among others, oxidative stress, which is a major factor in chronic low-intensity inflammation. In particular, visceral obesity and the endocrine functions of visceral adipose tissue, together with the secretion of inflammatory cytokines, are responsible for the main pathogenetic mechanisms underlying the development of weight-related NCD. A key factor is also insulin resistance, typical of the metabolic syndrome. This low-grade inflammatory status affects all organs and tissues and increases the risk of developing chronic degenerative diseases [7].

BMI is the benchmark used worldwide to measure excess body weight, although it has inherent limitations as a marker, mainly because it does not take into account body composition, and is not an indicator of excess fat mass, which is a defining characteristic of both overweight and obesity [8]. Higher-than-optimal BMI was estimated to cause 5 millions deaths worldwide from NCD [9, 10].

Obesity and overweight are increasing worldwide as well as NCD. The prevalence of obesity and overweight in the adult Italian population (18–69 years) in 2020–2021 was 10.4% and 32.5%, respectively, and it was higher among males, the trend has been stable for 13 years [11]. In 2022, the percentage of adults aged 18 + years with a body mass index (BMI) of 25 kg/m^2^ or higher was 49% in total (56% males and 42% females) [12]. These data are apparently in contrast with the healthy MD model that inspires the national food based dietary guidelines (FBDG). One possible explanation is the phenomenon of “nutritional transition”. Currently, low- and middle-income countries, as well as the richest ones, are witnessing this phenomenon mainly due to the Westernization of the diet, and a change in people’s eating habits. Urbanization has led to a shift from traditional foods to low-cost ultra-processed foods and, at the same time, technological development encourages a more sedentary lifestyle. One of the regions that is more involved in this transition is the area of Middle East [13], where the prevalence of obesity and overweight for people aged 15+ (based on studies from 2000 to 2020) in the MER was 21.2% and 33.1%, respectively [14]. In 2022 in Lebanon, the percentage of adults aged 18 + years with a BMI of 25 kg/m^2^ or higher was 65% (71% of males and 59% of females). In the occupied Palestinian territory (including east Jerusalem), the value was 69% (64% of males and 73% of females) [12], despite MD is the recommended dietary model in MERs too.

NCD data, about Italy, Lebanon and Palestine representative of the Western and MER countries, can depict the actual situation. The prevalence of CVD in 2019 in people of both genders aged 20 + years was higher in Italy (19.56% (95% C.I.: 18.77%; 20.33%)) compared to Lebanon (11.96% (95% C.I.: 11.41%; 12.49%)) and Palestine (7.74% (95% C.I.: 7.34%; 8.15%)). The prevalence of ischemic heart disease was higher in Lebanon (7.57% (95% C.I.: 7.02%; 8.14%)) than in Italy (5.63% (95% C.I.: 5.02%; 6.32%)) and Palestine (4.29% (95% C.I.: 3.96%; 4.63%)). The stroke had a higher prevalence in Lebanon (2.14 (1.99%; 2.32%)), followed by Italy (1.56% (95% C.I.:1.39%; 1.77%)) and Palestine (1.53% (95% C.I.:1.40%; 1.67%)). Diabetes mellitus of type 2, in Italy and for people of both genders aged 20+, in 2019 had a prevalence of 13.00% (95% C.I.: 11.57%; 14.43%)). In Lebanon, the value was 11.80% (95% C.I.: 10.57%; 13.19%)) and in Palestine 8.87% (95% C.I.: 8.01%; 9.67%)) [15].

MD is considered an excellent dietary pattern that can reduce the risk of obesity and overweight, and, consequently the related NCD, due to its high nutritional quality with beneficial health effects, from both the preventive point of view and as nutritional medical therapy. MD has the potential to be a sustainable dietary pattern in the future as well [16, 17]. Moreover, adherence to the MD has also been shown to be associated with a lower prevalence of metabolic syndrome [18] and lower incidence and mortality from CVD, as published in the “Seven Countries Study”. This study highlights how mortality due to coronary heart disease (CHD) was 2–3 times lower in Mediterranean countries. Mortality was significantly related to the amount of saturated fatty acids intake in the diet, and inversely related to the ratio of unsaturated to saturated fatty acids [19].

Based on this considerations, MD represents one of the best dietary models for all populations, respecting the local products and culinary habits. Therefore, the above-reported data concerning excess body weight and NCD, are apparently in contrast with the healthy MD model suggested by FBDGs, In fact, a recent study reveals unbalanced dietary habits among the Italian population, characterized by excess consumption of several foods (eggs, cooked fats, snacks, and sweets) and low consumption of fruits and vegetables [20].

The aims of the present study are addressed to find one possible explanation for the apparent paradox between the dietary recommendations provided through the FBDGs, respecting the characteristics of the MD, and the high prevalence of obesity, overweight, and NCD observed in these two regions. It is, therefore, necessary to consider: (i) the actual food intakes in Italy and MER, through literature data available; (ii) the possible association between different levels of adherence to MD with the prevalence of obesity and overweight in Italy and MER as assessed by BMI, through data available in the literature; (iii) finally, by implementing two 7-DFP, aligned with MD criteria, utilizing the typical dishes and dietary patterns of the two geographical regions under consideration, it could be possible to identify any deficiencies or excesses of specific nutrients to focus on, in order to reduce the risk of developing diet related NCD.

Concerning MER, this study was focused on Lebanon, the only country that has drafted FBDG in English, that are comparable to international FBDGs, including Italian ones. In addition, we extended the analysis to Palestine, which has similar food traditions and uses the same FBDG as a reference.

## Methods

### Assessing the association between different levels of adherence to the MD and BMI using the meta-analysis technique

#### Literature search

From October 2023 until November 2023, an extensive literature search was conducted in three electronic databases: PubMed, Scopus, and Embase. The search strings were modified accordingly for each electronic database and are presented in Supplementary Material 1.

The literature search included terms related to BMI, lipids (total cholesterol, HDL, LDL, and triglycerides), to analyze the association between them and the level of adherence to the MD.

### Study selection and eligibility criteria

We considered studies from the literature search that evaluated the association of adherence to the MD (divided into three levels: low, medium, and high), BMI, and lipids.

The three levels of adherence to the MD in each study were defined according to the validated questionnaire for assessing adherence, among others, KIDMED [21], and Mediterranean diet score [22].

The analyses were based on the outcomes in the three different levels of adherence to the MD and the absolute frequency of subjects in BMI classes. Regarding lipids, the mean and standard deviation of the levels of total cholesterol, LDL, HDL, and triglycerides according to the level of adherence to the MD were considered. Studies without this information were excluded from the analyses.

Given the limited studies conducted in Lebanon and Palestine, the research was extended to the entire MER.

We considered observational studies (cross-sectional and cohort studies) with healthy subjects and people who do not play sports at a competitive level. The literature search focused on studies published since 2005.

After the duplicate studies were removed, the relevance of studies on the topic of this analysis was assessed using a hierarchical approach based on the title, abstract, and full manuscript.

Only studies about adults (age 18+) were considered.

### Data selection and extraction

For each included study, the following information was extracted: record id (first author and publication year), study type (cross-sectional or cohort), data collection period, location, cohort name (in a case of cohort study), sample size (not necessarily it was the one declared in the study, but it was the number of subjects with information on outcome and the level of adherence to the MD), the age range of the study participants, gender of the participants (only females, only males or both gender), follow up period in case of the cohort study, diet evaluation method, MD adherence score used, subdivision criteria of MD adherence classes, number of subjects in each adherence classes, absolute frequency of subject of different BMI categories, across levels of adherence to the MD, or mean and standard deviation (or standard error) of cholesterol (total, LDL, HDL) and triglycerides across levels of adherence. Where reported, also the instrument of collection of data (i.e., how the BMI was assessed) were added.

The analysis regarding diabetes, cardiovascular disease, and lipids are not presented, because no publications satisfy the above inclusion criteria. The literature on those topics had data about the level of physical activity in the three levels of MD, which we considered because it is a protective factor against overweight and obesity. Analyses have been performed only on the outcomes about which we have publications from both countries that respect the inclusion criteria.

**Fig. 1:**
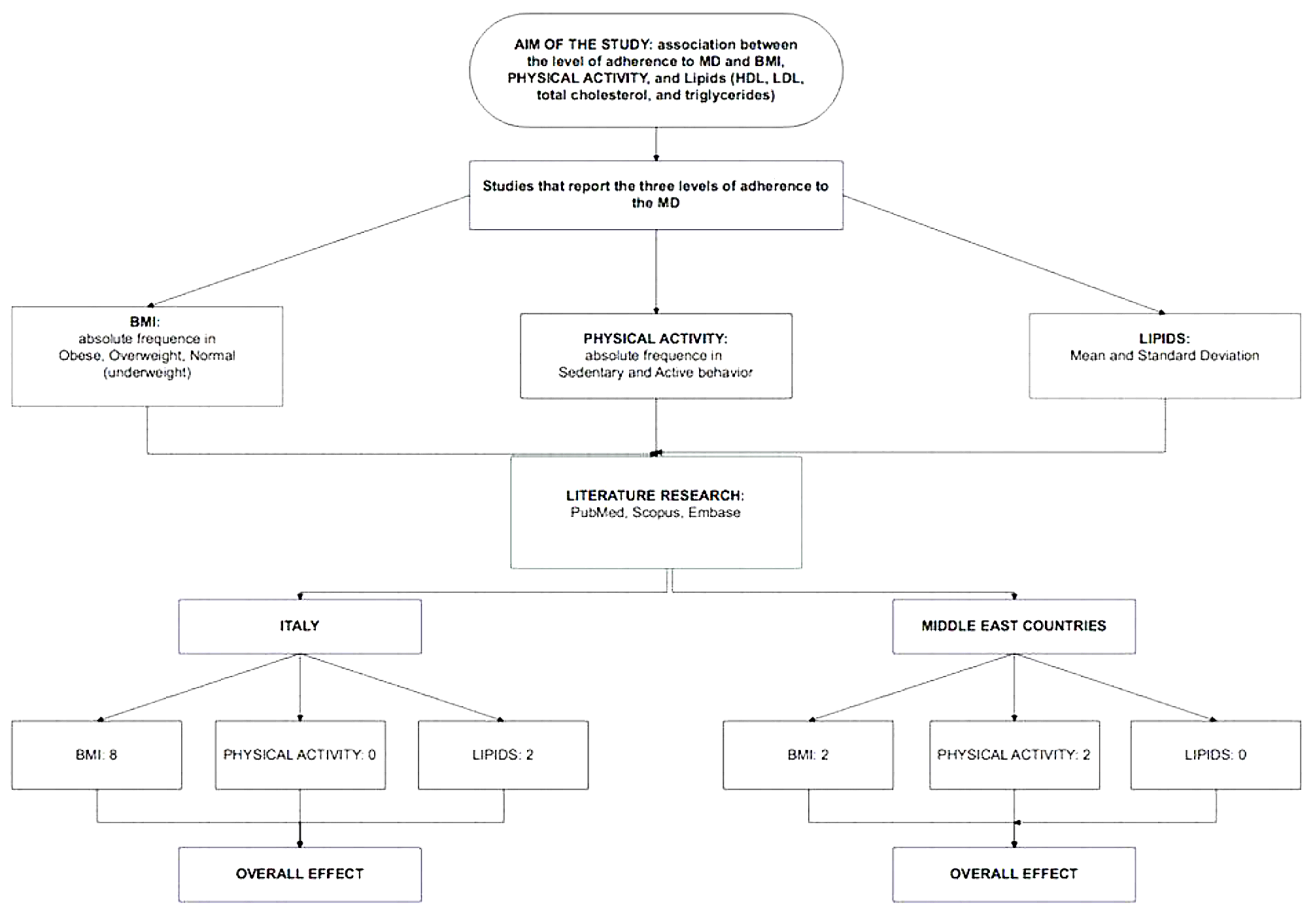
Flow chart for assessing the association between different levels of adherence to the Mediterranean diet and BMI, lipids, and physical movement

Figure 1 shows the flow chart for this section of methods.

### Statistical analysis

The aim was to estimate the overall effect of the associations between the three levels of adherence to the MD and BMI classes. Each study was weighted to combine results from different studies in a single overall effect with 95% Confidence Intervals. A random effect model was used to attribute the weight due to the expected heterogeneity.

Heterogeneity between studies was assessed using the τ ^2^, Cochrane Q test (where *p* < 0.1 was used as the significance level for evaluating the evidence of heterogeneity), and the I^2^ inconsistency index [23].

To this aim, the method of meta analysis was employed [24].

Results were reported by forest plot. For each study, a visual representation of the association of the BMI classes with MD adherence (in terms of OR) and its 95% Confidence Interval typically consists of a square whose dimensions are proportional to the weight of the study and a horizontal line that passes through the square. The pooled results of the analysis are represented by a diamond whose length is associated with the precision of the estimate. Vertical lines are added: one which represents the “absence of effect,” and one (dashed) represents the pooled results. The single study’s results that do not intersect the dashed line is an outlier [24].

Different analyses were performed for different locations (Italy or MER).

The odds ratio (OR) was used as the effect size for presenting the findings regarding classes of BMI, and the inference was based on the 95% Confidence Interval (95% CI) and the p-value.

In the analyses, two models were used: in the first one, the response variable was the proportion of obese subjects over normal BMI and underweight ones. The comparisons of proportion were the following: obesity in high adherence of MD vs. medium adherence to the MD; obesity in high adherence of MD vs. low adherence to the MD. The same comparisons were performed also for overweight classification over normal BMI and underweight ones. High adherence to the MD were considered the reference category.

The pooled odds ratio estimate was performed through the Mantel Haenszel method [25, 26]. The Restricted maximum likelihood estimator was used to calculate tau.

If outliers in the meta-analysis were present and the number of studies was more than 2, the outliers were removed, and the analysis was repeated.

The analyses were performed using the statistical program R software [27]. For the OR analysis, the metabin function of the meta package was used [28].

## Comparison among different FBDG

The latest publication of the Italian FBDG was published in 2018 [29] by CREA (Consiglio per la Ricerca in Agricoltura e l’Analisi dell’economia Agraria) Food and Nutrition Research Centre, which also published previous revisions in 1997 and 2003.

FBDG for the Lebanese population [30] was published by the Faculty of Agricultural and Food Sciences at the American University of Beirut in collaboration with the Lebanese National Council for Scientific Research (CNRS) in 2013. Moreover, the recipes used and analyzed in this study came from Palestine and Lebanon. Lebanon and Palestine (i.e., the territories of the Gaza Strip and West Bank), are included in the WHO MER classification [31].

### Implementation and comparison of Italian and MER 7-day dietary food plans

To compare the nutritional composition between the Italian and MER MD, the 7-DFPs were implemented by using fresh foods, regional recipes, and beverages for each dietary pattern, according to specific guidelines. The dishes were composed of different food groups (cereals, vegetables, meats, fish and sweets) according to recipes. Each 7-DFP was divided into three main meals (breakfast, lunch, dinner) and one or two snacks. The 7-DFP representing Italian eating habits was compiled according to the Italian FBDG [29].

The 7-DFP for the MER MD was compiled using Lebanese and Palestinian typical recipes provided by dieticians from Birzeit University (Palestine) or recipe books [32, 33]. The 7-DFP of each dietary pattern was elaborated meeting the nutritional requirements, in terms of macro- and micronutrients, of an adult with energy requirements of 2000 kcal/day. The two 7-DFP were analyzed using a software (MetadietaVR; METEDAsrl, via S. Pellico 4, San Benedetto del Tronto, AP, Italy). Metadieta software includes a vast database of food items with portion photos, constantly updated and implemented with values from the main official databases as: From Italy, Council for Agricultural Research and Agricultural Economics [34]; Also from Italy, BDA-IEO [35]; U.S. Deparment of Agriculture [36]; CIQUAL French Food Composition [37]. It is commonly used in the dietetic clinical practice inside hospitals and during outpatient activities, by dietitians and nutritionists. In the present work, it was specifically chosen MD adherent food items, respecting dietary habits and ensuring that the nutritional composition of meals reflected the usual pattern and recipes.

### Bromatological analysis of the Italian and MER 7-DFPs

For each day, the bromatological composition was analyzed in terms of proteins, carbohydrates, fiber, total fats, saturated fats, monounsaturated fats, polyunsaturated fats (grams); Eicosapentaenoic acid (EPA), Docosahexaenoic acid (DHA), cholesterol, sodium, calcium, iron, zinc, thiamine, riboflavin, niacin, vitamin C, vitamin E, vitamin B6 (milligrams); vitamin A retinol eq., folic acid, vitamin D, biotin, vitamin B12, vitamin K (micrograms). Based on the bromatological composition of the two 7-DFP, the mean and standard deviation (sd) were obtained for each nutrient. The weekly sum of each nutrient was also calculated, and the data were represented in graphs to allow comparison between macro and micronutrients of the two diets.

The bromatological composition of Italian and MER 7-DFP were compared.

The normality of the distribution of each macronutrient and micronutrient in the two groups was assessed through the qqplot and the Shapiro-Wilk normality test. When there was no evidence of a lack of normal distribution, the Student’t test, allowing for heteroscedasticity, was used (Welch Two Sample t-test). Results are reported as estimated differences between means, 95% Confidence Interval of this difference, test statistics, and p-value.

Otherwise, the non-parametric test of Wilcoxon-Mann-Whitney was employed. Results are reported by the Hodges-Lehmann estimator (i.e., the median of the difference between all the pairwise differences), with a 95% confidence interval [38].

The bromatological composition of the Italian and MER 7-DFPs were also compared to the specific recommendations for the intake of each nutrient (*LARN 2014/WHO-FAO*) [39, 40]. For this aim, the approximate 95% confidence interval of the mean, considering the daily intake of each nutrient of the bromatological composition, was calculated using the t distribution with 6 degrees of freedom.

The estimate was considered as too low or too high in the bromatological composition if the recommended value of the guidelines were out of the confidence interval.

**Fig. 2:**
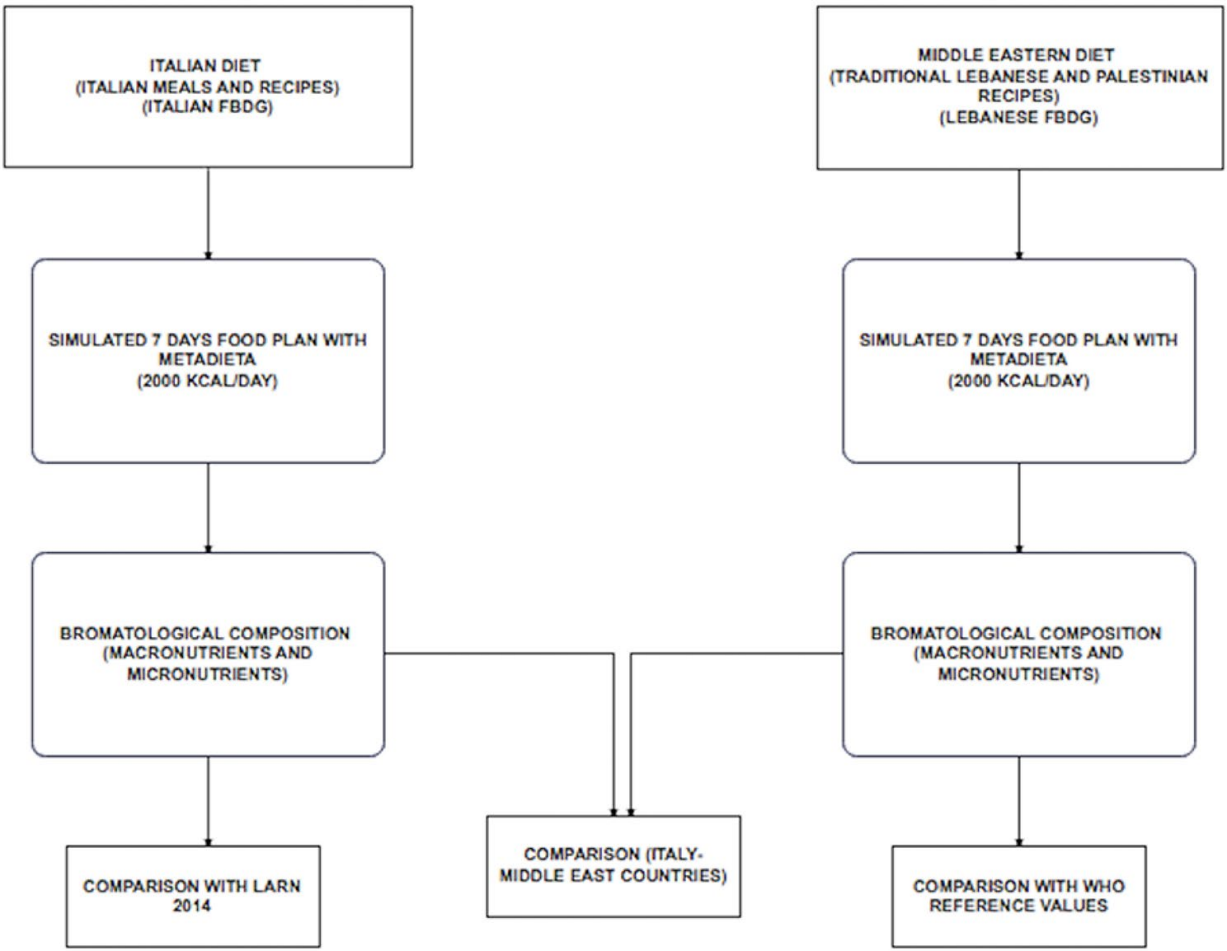
Flow chart for comparing the nutritional composition between the Italian and the MER 7-DFP

Figure 2 shows the flow chart for the implementation and comparison of Italian and MER 7-DFPs.

## Results

### Real food intakes in Italy and MER

For Italian population, IV SCAI survey reported an average energy intake for the population aged 3 to 74 years of 1933 kcal/day. There are no available data, from this report, on energy intake by age, particularly for the adult population. Considered overall, the energy breakdown is derived 15% from protein (5% from plant origin and 10% from animal origin), 42% from carbohydrates, and 34% from fat. The average intake of carbohydrates is 223 g/day, while that for starch and simple sugars is 127 g/day and 83 g/day, contributing on average 27% and 17% of total energy, respectively. The average fat intake is 82 g/day, including 26 g/day of saturated fatty acids contributing 12% of total energy, 38 g/day of monounsaturated fatty acids, corresponding to a contribution of 17% to total energy, and 11 g/day of polyunsaturated fatty acids, corresponding to 5%. Average daily fiber consumption is reported to be between 15 and 17 g/day [41].

However, it is estimated that Italians consume about + 75% of the needed daily caloric intake recommended [42].

As it concerns MER food intakes (Lebanon and Palestine), data from a meta-analysis conducted by Doggui et al. [43] report for overall MER: a mean energy intake of 2286.6 Kcal/day, 13.9% total energy from protein, 53.3% total energy from carbohydrates, and 32.2% total energy from fats. For the Lebanese population: 2680.9 Kcal/day, 11.8% of energy from protein, 42.2% from carbohydrates, 31.8% of energy from fats. Unfortunately, data for Palestine are not clear, due to some discrepancy on reporting calculations.

Moreover, another study on mean consumption of food by adults living in Beirut, estimated a mean energy intake of 2523.57 kcal/day. Fat contributed for 38.9% of total energy, protein 13.4% of total energy, and carbohydrates 47.2% of total energy [44]. In addition, there is another commonly used indirect way of estimating energy intake, which is based on consumption inferred from the food availability *per capita* in different countries. This estimate should take into account several corrective parameters, such as wastage and the fact that *per capita* food availability is strongly influenced by the economic conditions of the population groups considered.

Data from FAO [45] and Our World in Data [46] reported daily energy consumption of 3503 Kcal for Italian population and of 2857 Kcal for Lebanese population. No data are available for Palestinians.

### Adherence to MD and BMI

From Fig. 1 it is evident that the studies about BMI represent the only parameter to compare with adherence to MD in both Italy and MER, since no data are available about physical activity in Italy while no data area are available about lipids in MER.

Regarding the BMI, two studies were considered for MER: Panbehkar-Jouybari et al. [47] and El Hajj, Julien [48].

For the Italian area, eight studies were considered: Grosso G. et al. [49], Bonaccio M. et al. [50], Ricci E et al. [51], Ricci E et al. [52], Ruggiero E. et al. [53], Tessari S. et al. [54], Agnoli C. et al. [55], and Maugeri [56].

The characteristics of the studies for analyses of the association between BMI and adherence to the MD are reported in Luconi supplementary Table 1.

Based on the considered papers, the proportion of obese subjects among who have a high adherence to the MD is 17% in Italy and 31.1% in MER, among who have a medium adherence to the MD is 17.8% in Italy and 37.8% in MER, and among who have a low adherence to the MD is 16.6% in Italy and 32.1% in MER.

Concerning obesity, medium adherence to MD is a potential risk factor both for MER and Italy, in fact, ORs are all greater than 1. MER: 1.19 (95% C.I.: 0.99; 1.42) with a p-value of 0.062, Italy: 1.12 (95% C.I.: 0.90; 1.38), p-value = 0.311 with outliers and 1.01 (95% C.I.: 0.95–1.07), p-value = 0.71 when outliers were excluded from the analysis.

Those ORs are all greater than 1, their C.I. included one, and the p-values are greater than 0.05. The association is not statistically significant, thus there is no evidence that in the population the subjects with medium adherence to MD are more likely to be obese than subjects with high adherence to MD.

The heterogeneity between studies in MER was not statistically significant.

**Fig. 3:**
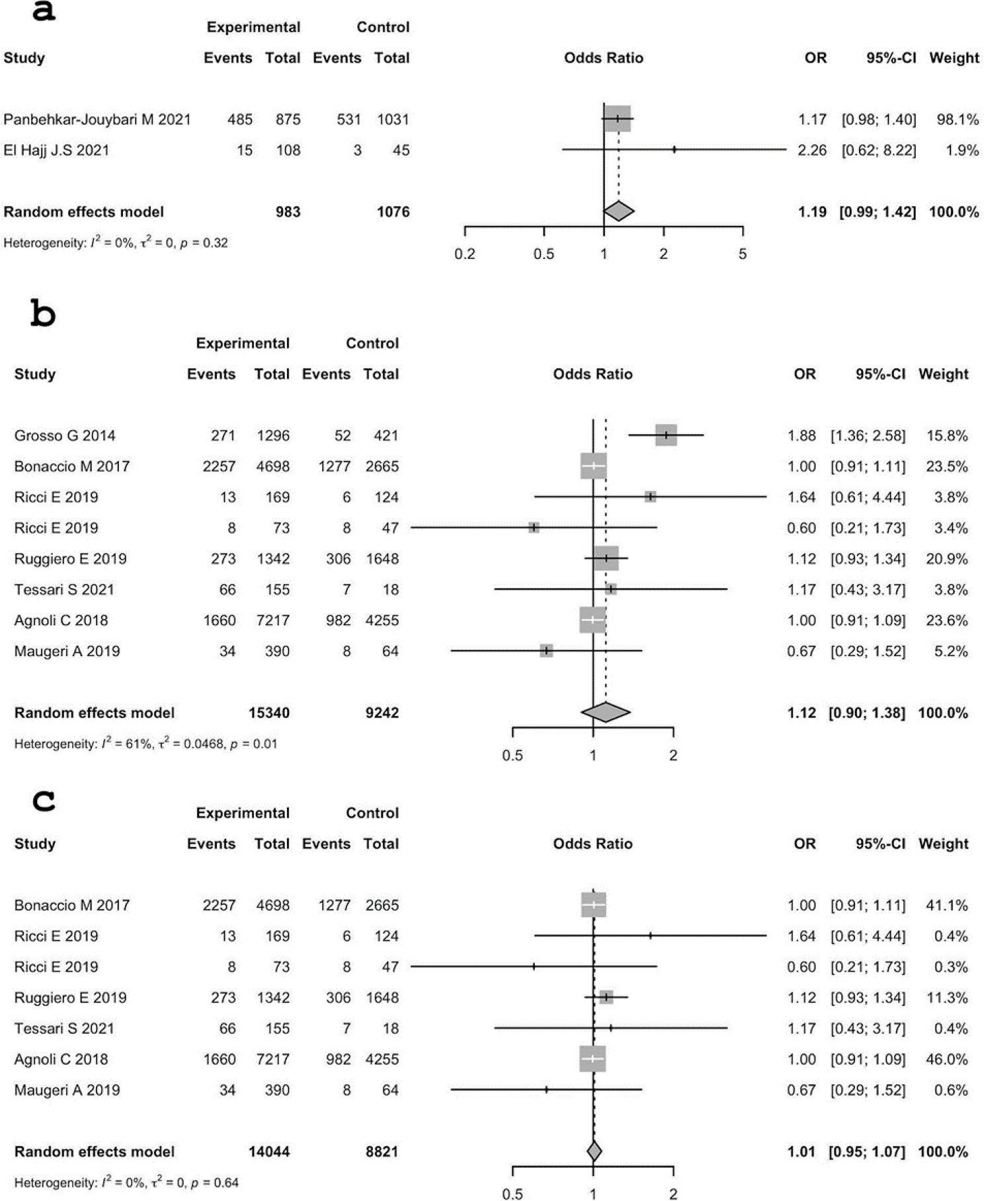
Analysis of the association between obesity and MD adherence (high vs. medium). **a**: MER, **b**: Italy, **c**: Italy without outlier

The heterogeneity between studies in Italy was statistically significant: (τ^2^ = 0.0468 (95% C.I.: 0.0024; 0.4734); I^2^ = 61.3% (95% C.I.: 16.2%; 82.1%); H = 1.61 (95% C.I.: 1.09; 2.36). Test of heterogeneity: Q = 18.08, d.f. 7, p-value = 0.012)). Results are presented in Fig. 3.

The same pattern can also be observed by comparing obesity in low adherence vs. high adherence. Low adherence seems to be a risk factor for obesity; in fact, all overall ORs are greater than 1, with a low value in MER (1.05 (95% C.I.: 0.88; 1.24) with a p-value = 0.598) and high value in Italy (1.42 (95% C.I.: 0.87; 2.31), p-value = 0.16). All ORs are not statistically significant, but when the outliers concerning Italy are removed, the OR is 1.20 (95% C.I.: 1.02; 1.41], p-value = 0.031, which is statistically significant. Thus, when studies with more homogeneous results are only considered, there is statistically significant evidence of low adherence to the MD as a risk factor for obesity in Italy.

The heterogeneity between studies in MER was not statistically significant.

**Fig. 4:**
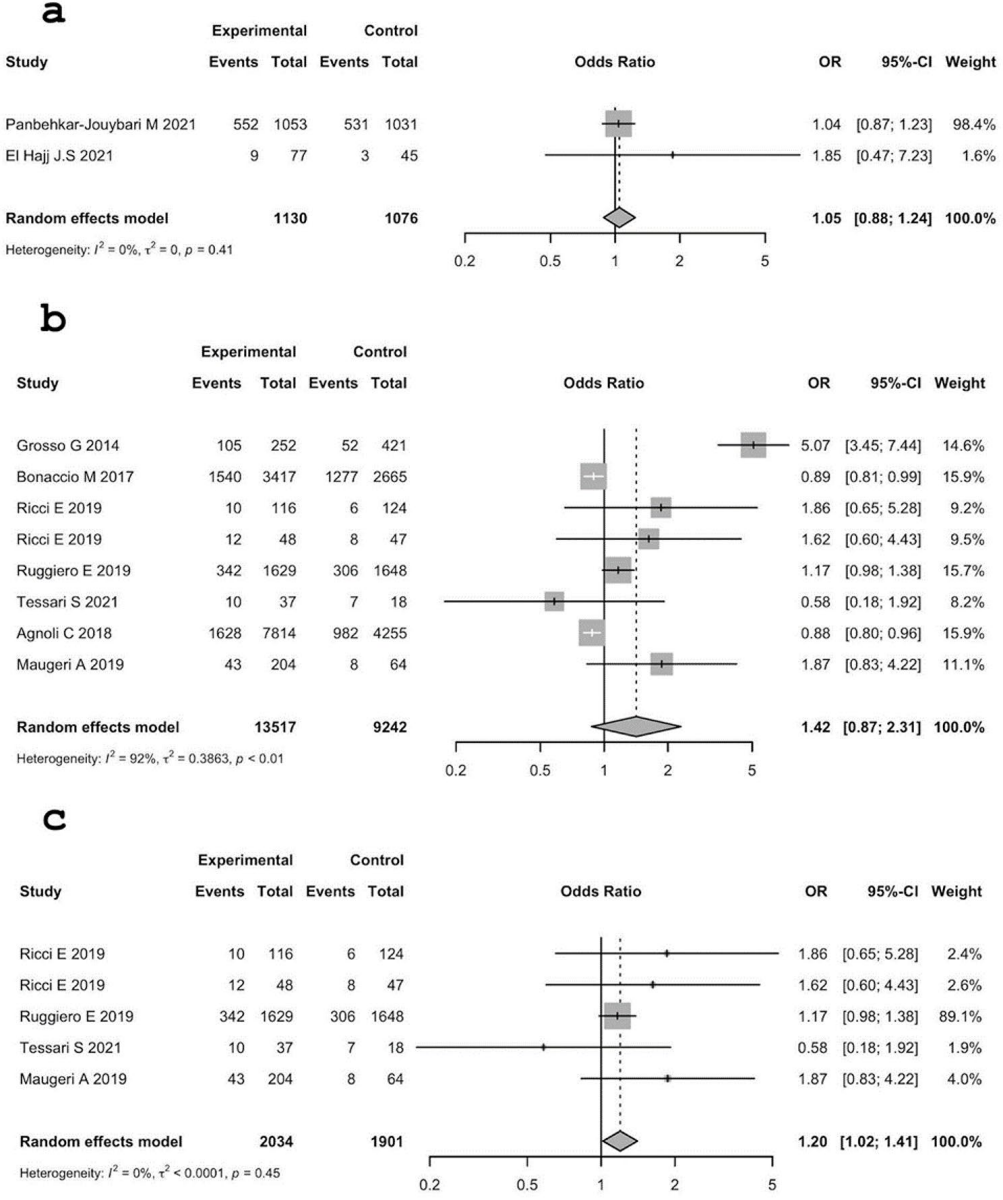
Analysis of the association between obesity and MD adherence (high vs. low). **a**: MER, **b**: Italy, **c**: Italy without outliers

The heterogeneity between studies in Italy with outliers was significant: τ^2^ = 0.3863 (95% C.I.: 0.1226; 1.6975); I^2 ^ = 92.1% (95% C.I.: 86.9%; 95.3%); H = 3.56 (95% C.I.: 2.76; 4.60), Q = 88.94, d.f. 7, p-value < 0.0001. Results are presented in Fig. 4.

Based on the considered papers; the proportion of overweight subjects among who have a high adherence to the MD is 40.6% in Italy and 37.4% in Middle East, among who have a medium adherence to the MD is 40.5% in Italy and in 25.7% Middle East, and among who have a low adherence to the MD is 39.1% in Italy and 35.3% in Middle East.

Concerning overweight, the association considering medium and high adherence to the MD is not in the same direction as previously described for obesity: ORs are less than 1, MER: 0.88 (95% C.I.: 0.38; 2.04) with a p-value of 0.763, Italy (without outliers): 0.99 (95% C.I.: 0.95–1.04], p-value = 0.725. Those results suggest that medium adherence to the MD is not a risk factor for overweight.

The heterogeneity among the study in MER was statistically significant: tau^2 = 0.3053; I^2 = 80.0% (95% C.I.: 13.8%; 95.3%); H = 2.23 (95% C.I.: 1.08; 4.63), Q = 4.99, d.f = 1, p-value = 0.0255.

**Fig. 5:**
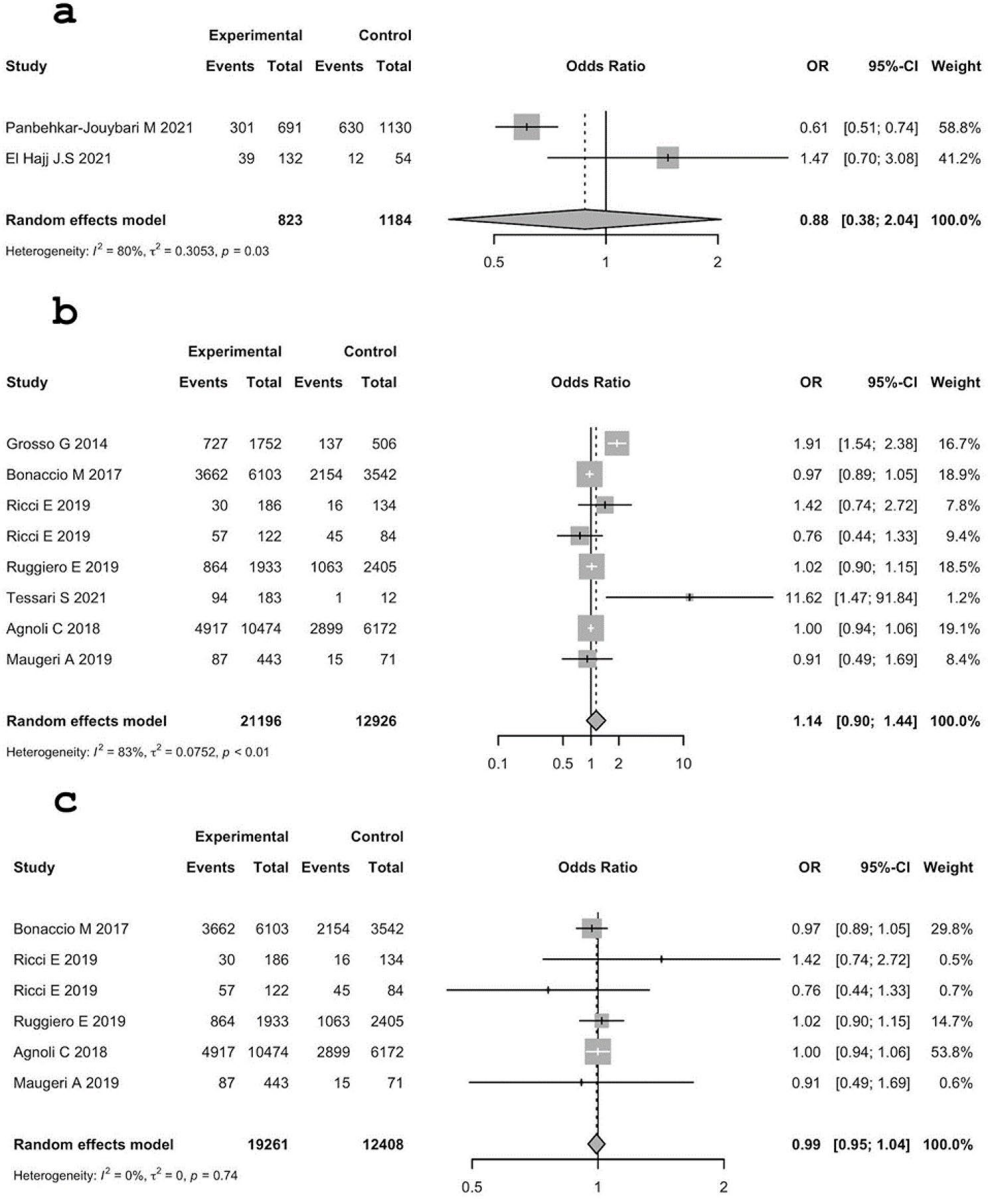
Analysis of the association between overweight and MD adherence (high vs. medium). **a**: MER, **b**: Italy, **c**: Italy without outlier

The heterogeneity between studies in Italy was statistically significant: tau^2 = 0.0752 (95% C.I.: 0.0258; 2.3834); I^2 = 83.1% (95% C.I.: 68.0%; 91.0%); H = 2.43 (95% C.I. 1.77; 3.34). Q = 41.31, d.f. 7, p-value: < 0.0001. Results are presented in Fig. 5.

Concerning the association of overweight in high vs. low adherence to the MD, the results of the studies are very heterogeneous: some of them have ORs greater than 1, others less than 1. Thus, the results seem to be contradictory. The overall OR for MER is 0.95 (95% C.I.: 0.81; 1.12) with a p-value of 0.524. In Italy, the overall OR is 1.39 (95% C.I.: 0.85;2.27), p-value = 0.194. The overall estimates in MER and Italy suggested different patterns: low adherence in MER seems not to be a risk factor for overweight; on the contrary, in Italy, low adherence seems to be a risk factor for overweight.

The heterogeneity between studies in MER is not statistically significant.

**Fig. 6:**
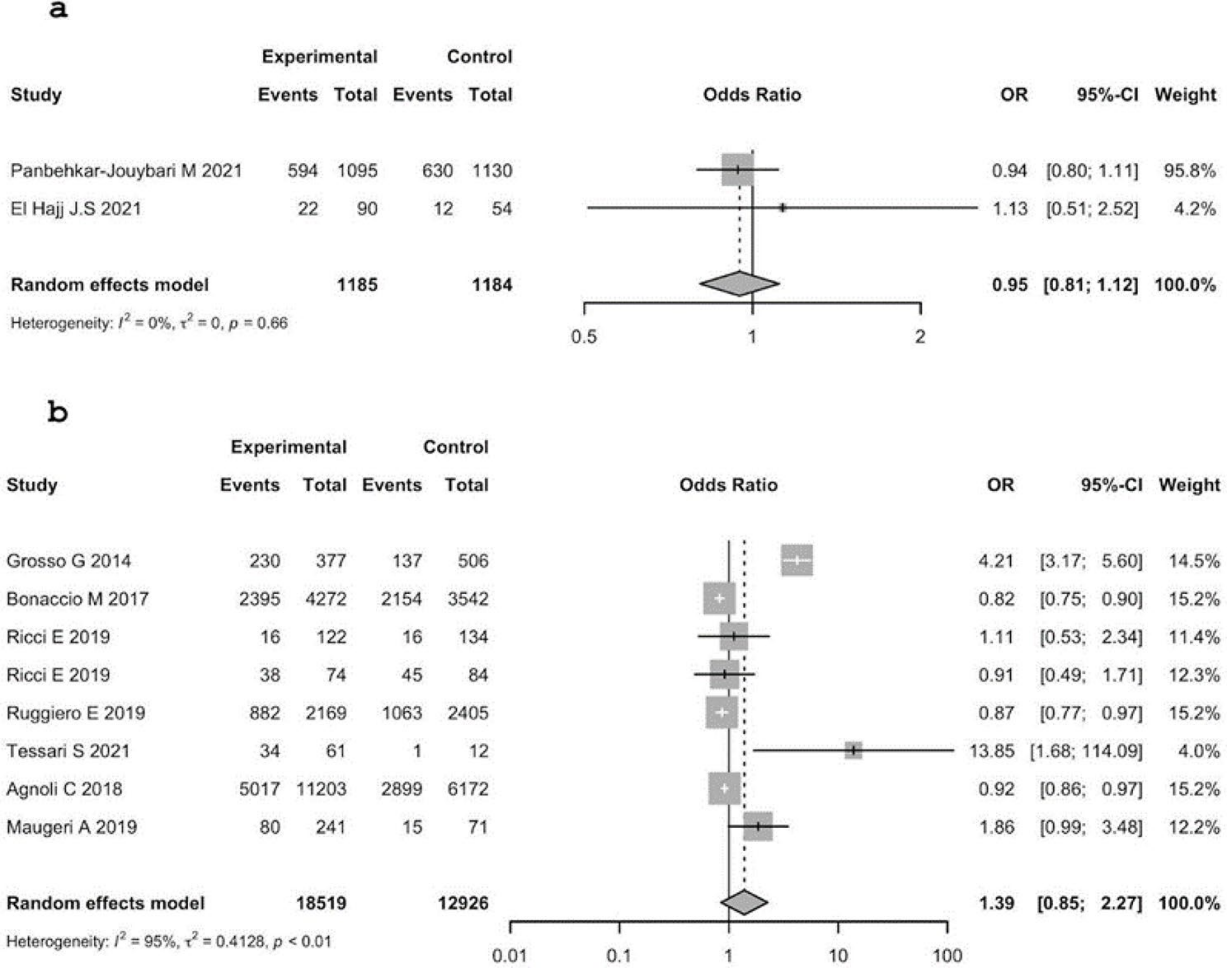
Analysis of the association between overweight and MD adherence (high vs. low). **a**: MER, **b**: Italy

The heterogeneity between studies in Italy is statistically significant: tau^2 = 0.4128 (95% C.I.: 0.1596; 3.5710]; I^2 = 94.5% (95% C.I.: 91.3%; 96.5%); H = 4.27 (95% C.I.: 3.39; 5.38), Q = 127.78, d.f. 7, p-value < 0.0001. There were too many outliers to conduct an analysis without them. Results are presented in Fig. 6.

#### Comparison among different FBDG

Supplementary Tables 2 and 3 report the topics covered by the Italian Dietary Guidelines for Healthy Eating and Lebanese Adults, respectively.

Both FBDG underline the importance of controlling body weight through an active life besides nutrition. The protective effect of fruits, vegetables, legumes, whole grains, and omega-3 fatty acids (FA) and the associated risk of excessive consumption of salt, sugars, and saturated fats constitute the same main recommendations. Alcohol consumption is not mentioned in Lebanese FBDG as well as nutritional supplements. Recommendations for specific categories (Special tips) like pregnant women, the elderly, and vegetarians are contained in a dedicated chapter in Italian FBDG. In the Lebanese one, specific indications are present in sections in the individual chapters. As in the Italian dietary guidelines, in the Lebanese ones, the issue of food safety is addressed, while the issue of diet sustainability is not mentioned.

To better explain these key points, and to follow a varied diet the foods are classified into groups, and advice is given on the frequency with which to consume different foods from each group, as well as the recommended portions for each food according to daily energy requirements (Tables 1 and 2).

**Table 1:** Italian dietary recommendations for an energy requirement of 2000 kcal per day. Frequency of consumption of the different foods within each food group and recommended servings for a 2000 kcal daily diet

Food groups	Servings	Example of one serving
grains and grain-products, tubers	• Bread: 3.5 servings per day • Pasta and cereals: 1.5 servings per day • Bread substitutes: 1 per week • Sweet baked products: 2 per week • Breakfast cereals: 2 per week	• 1 Slice of loaf bread • 4 Tablespoons rice, barley, or brown • 3–4 Rusks • 2–3 Biscuits • 6–8 Tablespoons of cornflakes
Fruit and vegetables	• Fresh fruit: 3 servings per day • Unsweetened dried fruit: 3 servings per day • Fresh vegetables: 2.5 servings per day • Leaf salad: 2.5 servings per day	• 1 Medium/2 small fruits • 3 Apricots, 2 tablespoons of raisins • 2–3 Tomatoes, 3–4 carrots • 1 Large bowl of lettuce
Meat, fish, eggs and legumes	• Red meat: 1 serving per week • White meat: 2 servings per week • Fish: 2 servings per week • Eggs: 3 servings per week • Legumes: 3 servings per week	• 1 Beef slice • 1 Slice of chicken or turkey • 1 Little fish, 20 shrimps • 1 Medium egg • Half a plate of fresh legumes 3–4 Tablespoons dried legumes
Milk and dairy products	• Milk, yogurt, fermented milk: 3 servings per day • Cheese: 3 servings per week	• half a cup of milk, 1 jar of yogurt • 100 g of light cheese, 50 g of fat cheese
oils and fats for seasoning	• Vegetable oils: 3 servings per day • Animal or vegetable fats: 3 servings per day • Nuts, seeds: 2 servings per week	• 1 Tablespoon of olive oil • 0.5 Knob of butter, 10 g of cream • 7–8 nuts, 3 tablespoons of seeds

**Table 2:** Lebanese dietary recommendations for an energy requirement of 2000 kcal per day. Frequency of consumption of the different foods within each food group and recommended servings for a 2000 kcal daily diet

Food groups	Servings	Example of one serving
Cereals and grains	6 servings per day (at least 1/2 whole-grain)	• ¼ big loaf of Arabic whole-wheat pita bread • 1 slice of whole-wheat loaf (toast) bread • ½ cup cooked ‘Burghul’, wheat, rice, pasta • 1 cup ready-to-eat breakfast cereal (unsweetened)
Fruit	2 servings per day	• 1 small apple • 1 large banana, orange, or peach • ½ cup dried fruit • 1 cup fresh fruit juice
Vegetables	2–3 servings per day	• 1 cup raw vegetables • 2 cups raw green leafy vegetables • 1 cup cooked vegetables • 1 cup vegetable juice
Low-fat milk and dairy products	3 servings per day	• 1 cup liquid milk or yogurt • 3 tablespoons powdered milk • 45 g white cheese •1 cup milk-based pudding (‘Mhalbiyeh’, ‘Sahlab’ or ‘Riz Bi Halib’) • 8 tablespoons ‘Labneh’
Protein-rich foods	5-6.5 servings per day	• 30 g cooked lean red meat or white meat (poultry or fish) • 1 whole egg or 1.5 egg whites • ¼ cup legumes • 15 g unsalted nuts or seeds

Although the recommended frequency of meat consumption is higher than the Italian guidelines, the Lebanese FBDG recommends consuming fish products at least twice a week and varying other protein sources daily. If fish products are unavailable, it is recommended to consume fish oil supplements or plant sources of omega-3 fatty acids, such as walnuts and flaxseeds. The consumption of poultry and lean meat is recommended to address micronutrient deficiencies since the prevalence of iron, vitamin B12, and zinc deficiency is still high in the Lebanese population. Consumption of low-fat dairy products is also strongly recommended, especially as a source of vitamin D and calcium for bone health.

The use of graphic representations allows the population to understand the messages of the dietary guidelines quickly and easily.

The food pyramid is a widely used type of representation. It was designed and used, since 1992, in the United States. The food pyramid was joined to the healthy plate in 2011 [57]. In the case of the pyramid, the foods that should be consumed most frequently are at the base, while the voluptuous foods are at the top. The plate, on the other hand, represents the ideal components of a meal, and the size of the wedges indicates their mutual quantity [29]. Italian FBDG has been graphically illustrated by the Italian pyramid which presents the water consumption in the base, then three distinct parts indicate daily, weekly, and occasional consumption, each of which reports the serving sizes.

A cedar tree (a symbol also featured on the country’s national flag) is used in Lebanese FBDG, whose foliage represents the food groups (at the base those to be consumed most abundantly and those at the top to be limited). The trunk represents the water to be consumed daily. Also depicted in the background is the recommendation of physical activity through two people jogging [30].

### Different meal structure and calorie distribution in Italy and MER

There are significant differences in the distribution and composition of meals relative to Italian and MER eating habits. Breakfast is the meal with the greatest differences; in fact, breakfast in Italy often does not cover the recommended daily calorie share (15–20% of total calories), and more than 10% of Italians do not eat breakfast. The MER breakfast differs greatly from the Italian breakfast, and includes eggs, with traditional bread, *labne* and cheese. According to ISTAT data, most Italians (69.1%) consider lunch the main meal, although the percentage of individuals making dinner the main meal has increased in recent decades [58].

Dining in Lebanon and Palestine is varied and influenced by Arab Muslim tradition, with lamb predominating, and abundant use of nuts, fruits and vegetables included in the dishes. Lunch is introduced by meze, such as *hoummus bi-tahineh* (chickpea puree and sesame paste) baba *ghanooge* (roasted eggplant puree) and *falafel* (dried fava bean balls) cheeses, sauces and vine leaves stuffed with rice, those appetizers are accompanied by traditional bread. Soups often contain lentils and spices. A meat or fish dish with rice and shell fruits follows the appetizers; the side dish is salads such as tabouleh, which contains bulgur and various fresh vegetables. The meal ends with a dessert that is very sweet and rich with dried fruits and honey mase syrup. The drinks are *ayran* (made from sweet or salty yogurt) and *jellab* made from raisins). Although it is difficult to describe a “typical” Italian lunch one prototype consists of a pasta dish topped with tomato sauce, a meat or fish main course and a side of vegetables, followed by fruit.

Dinner often becomes the main meal, shared with the family, since lunch is often eaten outside the home for work-related matters. It is therefore common for lunch and dinner to be reversed.

Recent studies highlight that modern Italian diets deviate significantly from traditional Mediterranean dietary patterns. Contemporary Italian diets may feature an excessive intake of certain foods like eggs and cooked fats, diverging from the Mediterranean diet’s emphasis on balance and plant-based foods [58–60].

An example of one day of both Italian and MER 7-DFP is reported in supplementary Table 4.

### Macro- and micronutrient composition and differences between Italian and MER 7-DFPs

Table 3 shows the comparison between the macronutrients and micronutrients in terms of bromatological composition derived from the 7-DFP analysis for the Italian diet and MER.

**Table 3:** Comparison between the macronutrients and micronutrients in terms of bromatological composition derived from the 7-DFP analysis for the Italian diet and MEd. Legend: ME = Middle Eastern, sd = standard deviation, Mean difference: difference between Italy and ME, C.I. (Confidence Interval) of the difference. The difference in location is the median of the difference between all the pairwise differences. *it was not possible to calculate the exact p-values because of the presence of ties

MACRONUTRIENTS: T test (Welch Two Sample t-test)	Mean Italian 7- DFP (sd)	Mean ME 7- DFP (sd)	Mean difference	95% C.I.	p-value
Energy (kcal)	1997.43 (30.31)	2032 (36.38)	-34.57	-73.71; 4.57	0.078
Protein (g)	79.99 (6.56)	78.31 (11.13)	1.68	-9.25; 12.60	0.739
Total carbohydrate (g)	277.28 (27.58)	265.32 (20.7)	11.96	-16.68; 40.61	0.378
Saturated Fat (g)	18.07 (4.51)	19 (3.19)	− 0.93	-5.53; 3.68	0.666
Polyunsaturated Fat (g)	11.1 (4.21)	8.9 (1.52)	2.2	-1.75; 6.14	0.233
**MACRONUTRIENTS: Wilcoxon-Mann-Whitney test**	**Median Italy (first quartile, third quartile)**	**Median ME 7- DFP** **(first quartile,third quartile)**	**Difference** **in location**	**95% C.I of difference in location**	**p-value**
Total Fat (g)	65.25 (59.91, 70.73)	76.69 (75.08, 79.84)	-10.36	-20.51; -2.00	0.017
Sugar (g)	75.86 (71.55, 77.27)	66.98 (62.55, 85.56)	-1.7	-24.05; 15.17	> 0.99
Fiber (g)	32.85 (32.00, 32.92)	22.15 (20.21, 25.71)	10.7	4.23; 12.88	0.026
Cholesterol (mg)*	96.25 (96.25, 117.72)	145.0 (100.8, 292.6)	-40	-226.10; 96.25	0.304
Monounsaturated Fat (g)*	29.06 (28.70, 29.65)	45.87 (41.12, 47.05)	-15.88	-18.92; -7.27	0.0049
EPA (mg)*	0 (0.00, 0.07)	0 (0.00, 0.02)	5.19*10^− 5^	-0.01; 0.09	0.671
DHA (mg)*	0 (0.00, 0.07)	0 (0.00, 0.04)	8.98*10^− 5^	-0.04; 0.12	> 0.99
**MICRONUTRIENTS: T test (Welch Two Sample t-test)**	**Mean Italian 7- DFP** **(sd)**	**Mean ME 7- DFP (sd)**	**Mean** **difference**	**95% C.I.**	**p-value**
Calcium (mg)	829.43 (159.7)	631.87 (242.44)	197.56	-45.71; 440.84	0.101
Sodium (mg)	1692.40 (24)	1028.12 (431.6)	664.28	244.42; 1084.15	0.006
Iron (mg)	11.75 (1.66)	11.10 (4.7)	0.66	-3.74; 5.06	0.736
Zinc (mg)	10.34 (2.26)	8.61 (2.76)	1.74	-1.21; 4.69	0.223
Thiamine (mg)	1.47 (0.48)	1.02 (0.41)	0.45	-0.07; 0.97	0.086
Niacin (mg)	20.12 (4.17)	15.34 (6.88)	4.78	-2.01; 11.56	0.148
Vitamin A eq. retinol (µg)	1224.71 (825.83)	1194.53 (702.49)	30.18	-865.23; 925.59	0.943
Vitamin C (mg)	249.56 (97.63)	139.52 (60.52)	110.04	13.33; 206.75	0.030
Vitamin E (µg)	11.29 (2.79)	13.82 (2.28)	-2.53	-5.51; 0.45	0.089
Folic acid (µg)	502.07 (186.76)	397.14 (149.12)	104.94	-92.95; 302.82	0.269
Vitamin B6 (mg)	2.35 (0.43)	2.30 (0.69)	0.05	-0.63; 0.73	0.877
Biotin (µg)	17.06 (3.79)	20.50 (6.98)	-3.44	-10.20; 3.32	0.28
Vitamin B12 (µg)	2.96 (1.98)	2.52 (1.5)	0.44	-1.63; 2.50	0.651
**MICRONUTRIENTS: Wilcoxon-Mann-Whitney test**	**Median Italy (first quartile, third quartile)**	**Median ME 7- DFP** **(first quartile,third quartile)**	**Difference** **in location**	**95% C.I of difference in location**	**p-value**
Riboflavin (mg)	1.73 (1.67, 1.81)	1.51 (1.37, 1.62)	0.24	-0.03; 1.16	0.073
Vitamin D (µg)*	0.61 (0.31, 2.25)	0.46 (0.3, 1.31)	0.11	-0.86; 3.10	0.798
Vitamin K (µg)*	6.15 (5.47, 15.14)	5.23 (2.26, 5.76)	4.95	-3.94; 15.96	0.201

The main results highlight (i) The values of the total fats, according to the 7-DFP for MER tend to be significantly higher than the values of the Italian 7-DFP (difference in location=-10.36). (ii) The values of the fiber in the Italian 7- DFP tend to be significantly higher than those of the Middle Eastern diet (Med) (difference in location = 10.7). (iii) The values of Monounsaturated Fat according to the 7-DFP MEd tend to be significantly higher than those in the Italian one (different in location=-15.88). IV) The mean of sodium of 7-DFP in Italy is significantly higher than the one referred to the 7-DFP Med. V) The mean of vitamin C according to 7-DFP in Italy is significantly higher than that of Med.

**Fig. 7:**
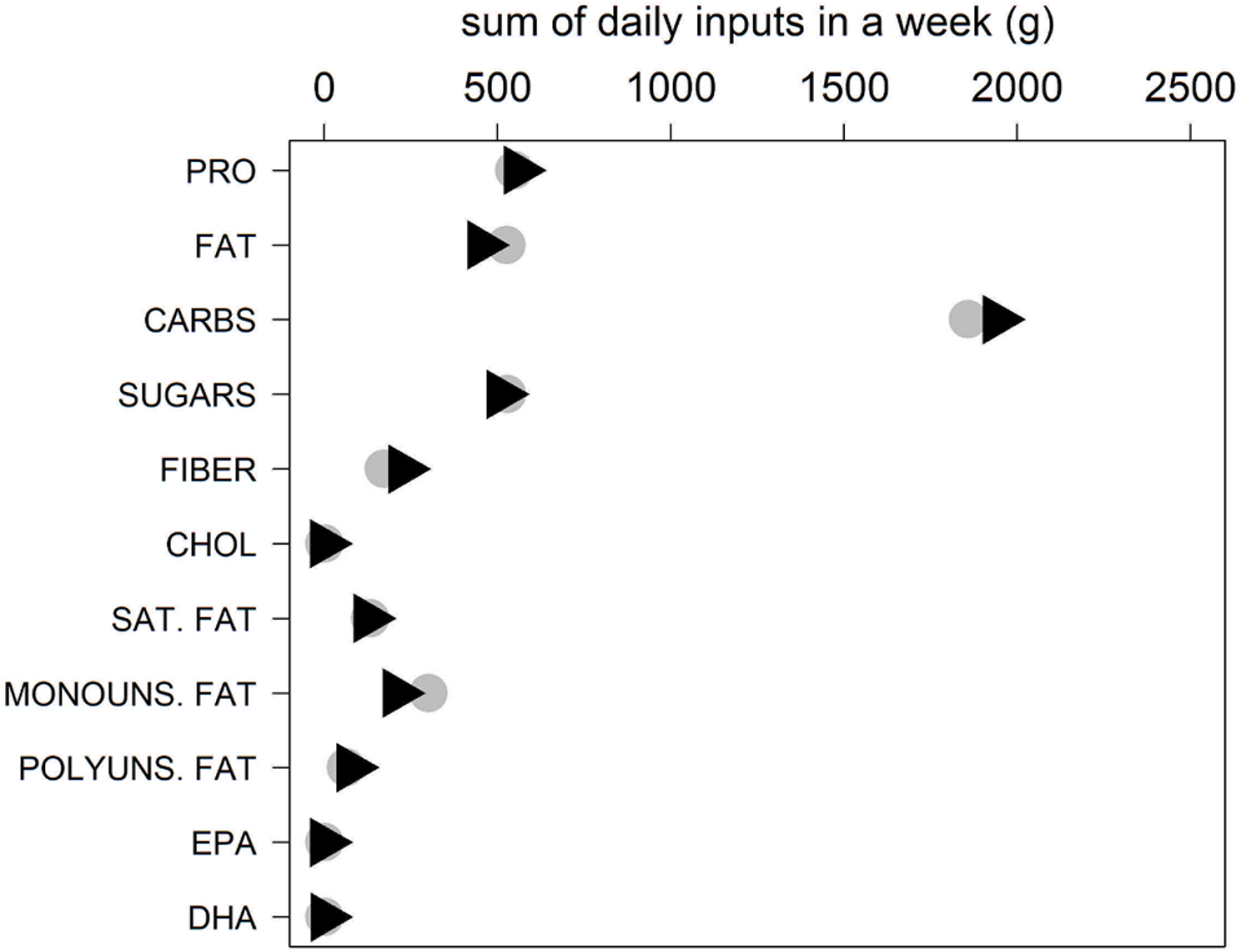
Daily macronutrient intake in a week in Italy and the MERs. Sum of daily macronutrients in a week in Italy (black triangles) and the MER (grey circles): protein (PRO), total fat (FAT), total carbohydrate (CARBS), total sugars (SUGARS), dietary fiber (FIBER), cholesterol (CHOL), saturated fat (SAT. FAT), monounsaturated fat (MONOUNS.FAT), polyunsaturated fat (POLYUNS. FAT), EPA, DHA. Inputs are expressed in grams

**Fig. 8:**
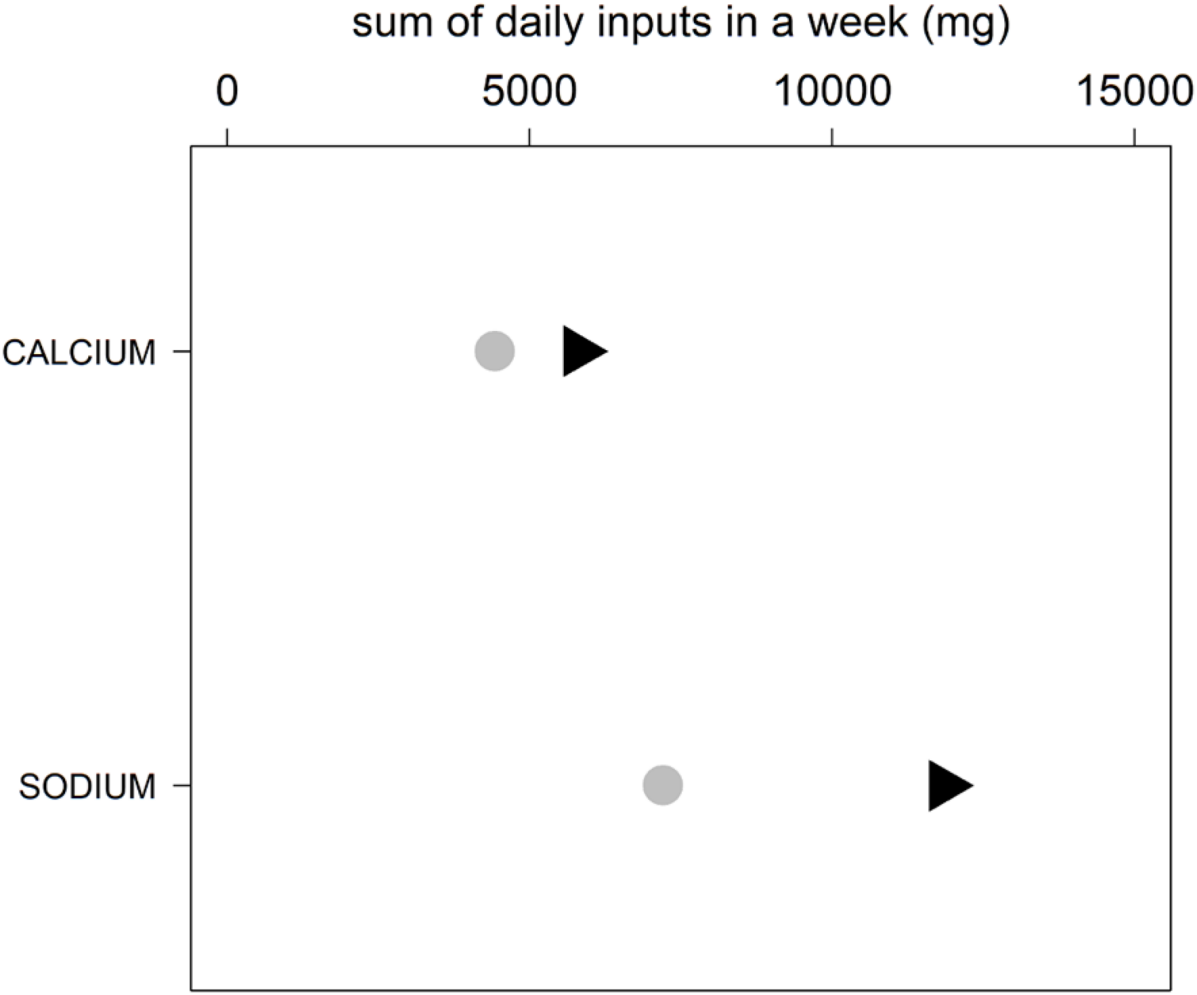
Daily calcium and sodium intake in a week in Italy and MER. Sum of daily micronutrients in a week in Italy (black triangles) and MER (grey circles): CALCIUM and SODIUM. Inputs are standardized in milligrams

**Fig. 9:**
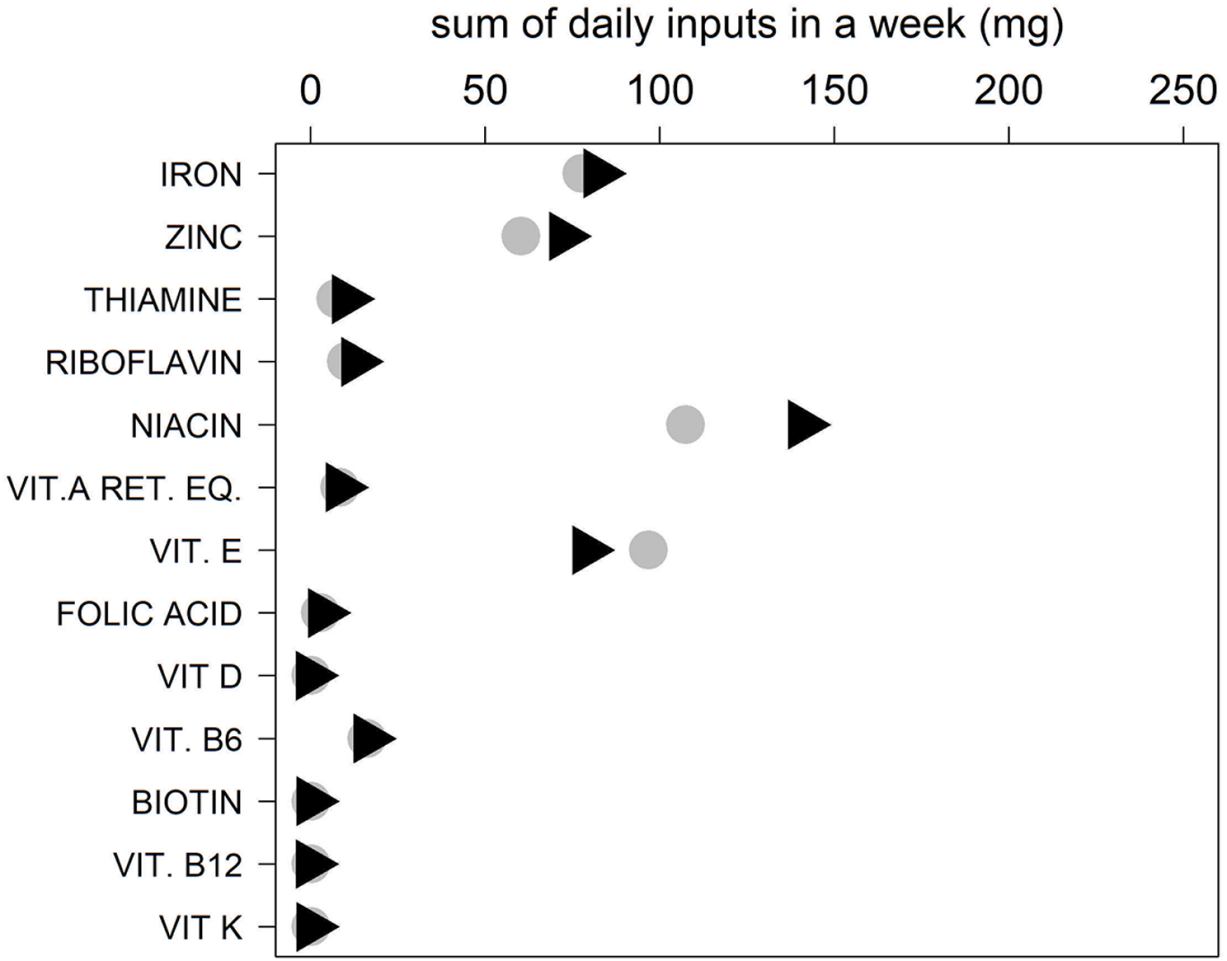
Sum of daily micronutrients in a week in Italy (black triangles) and MER (grey circles): iron, zinc, thiamine, riboflavin, niacin, vitamin A retinol equivalent (vitamin A eq. retinol), vitamin E (vit. E), folic acid, vitamin D (vit. D), vitamin B6 (vit. B6), biotin, vitamin B12 (vit. B12), vitamin K (vit. K). Inputs are standardized in milligrams

The total weekly macro- and micronutrients intakes of the two dietary models are shown in Figs. 7, 8, and 9, which show a major difference between the Italian and Middle Eastern dietary patterns in the weekly intakes of sodium, while the intake of calcium, iron, vitamin A, folic acid, vitamin D, vitamin B6, biotin, vitamin B12, thiamine, riboflavin, are very similar in both diets.

The bromatological composition of the 7-DFP of the Middle Eastern countries and Italy was compared to the recommendations for the intake of each nutrient in their respective guidelines. Results are reported in Table 4.

**Table 4:** Comparison between the recommendation for daily intake of each nutrient in MER and Italy and the bromatological composition of the 7-day diets. *the value is rounded as zero when the lower value of CI was below zero. Italy Mean: mean value calculated in a day based on one week in Italy, MER Mean = mean value calculated in a day on the basis of one week in MER, C.I.= Confidence Interval. M = male; F = female; E = Energy (2000 kcal). n.d.: not determined

NUTRIENT	Italy Mean (95% C.I.)	MER Mean (95% C.I.)	Recommended intake by LARN 2014 [39]	Recommended intake by WHO/FAO [40]
**Energy (kcal)**	1997.43 (1969.39; 2025.46)	2032.00 (1998.35; 2065.65)	2000 kcal	2000 kcal
**Proteins (g)**	79.99 (73.92; 86.06)* 16 E%	78.31 (68.02; 88.61) * 15.7 E%	0.9 g/bw (63 g considering an adult of 70 kg) 10–15%E	0.83 g/bw **(**58.1 g considering an adult of 70 kg)
**Carbohydrates (g)**	277.28 (251.77; 302.79) 55.5%E	265.32 (246.18; 284.46) 53.1%E	45–60 E% (225–300 g)	55–75 E% (275–375 g)
**Sugars (g)**	72.88 (65.99; 79.76) 14.2%E	75.25 (59.64; 90.86) 12.6%E	< 15 E% (80 g)	< 10 E% (53 g)
**Fibers (g)**	32.43 (31.76; 33.10)	24.66 (17.59; 31.74)	> 25 g	> 25 g
**Cholesterol (mg)**	123.82 (70.62; 177.02)	180.56 (61.30; 299.81)	< 300 mg	< 300 mg
**Total fats (g)**	64.87 (57.64; 72.10) 29.4 E%	75.11 (67.78; 82.45) 34.5 E%	20–35 E% § (44–78 g)	15–30 E% (33–67 g)
**Saturated Fat (g)**	18.07 (13.90; 22.24) 8.1%E	19.00 (16.05; 21.95) 8.6%E	< 10 E% (< 22 g)	< 10 E% (< 22 g)
**Polyunsaturated Fat (g)**	11.10 (7.20; 14.99) 5 E%	8.90 (7.49; 10.30) 4 E%	5–10 E% (11–22 g)	6–10 E%, (13–22 g)
**EPA (g)** **(mg)**	0.24 (0*; 0.76) 240 (0-760)	0.02 (0*; 0.04) 20 (0–40)	250 mg	200 mg
**DHA (g)** **(mg)**	0.31 (0*; 1.01) 310 (0; 1010)	0.04 (0*; 0.12) 40 (0; 120)	250 mg	200 mg
**Calcium (mg)**	829.43(681.73; 977.13)	631.87(407.65; 856.08)	1000 mg	1000 mg
**Sodium (mg)**	1692.40 (1469.51, 1915.29)	1028.12 (628.96, 1427.28)	1500 mg	2000 mg
**Iron (mg)**	11.75 (10.22; 13.29)	11.10 (6.75; 15.44)	10 mg (M) 18 − 10 mg (F)	11 mg
**Zinc (mg)**	10.34 (8.25; 12.44)	8.61 (6.05; 11.16)	12 mg (M) 9 mg (F)	11 mg
**Thiamine (mg)**	1.47 (1.03; 1.91)	1.02 (0.64; 1.40)	1.2 mg (M) 1.1 mg (F)	1.2 mg (M) 1.1 mg (F)
**Riboflavin (mg)**	1.84 (1.49; 2.19)	1.46 (0.94; 1.97)	1.6 mg (M) 1.3 mg (F)	1.3 mg (M) 1.1 mg (F)
**Niacin (mg)**	20.12 (16.27; 23.97)	15.34 (8.98; 21.71)	18 mg	16 mg (M) 14 mg (F)
**Vitamin A eq. retinol (µg)** **(mg)**	1224.71 (460.94; 1988.47) 368 (138; 597)	1194.53 (544.83; 1844.23) 359 (164; 554)	700 µg (M) 600 µg (F)	600 (mg RE, M) 500 (mg RE, F)
**Vitamin C (mg)**	249.56 (159.26; 339.85)	139.52 (83.55; 195.49)	105 mg (M) 85 mg (F)	45 mg
**Vitamin E (µg)**	11.29 (8.71; 13.88)	13.82 (11.71; 15.93)	13 µg (M) 12 µg (F)	13 mg (M) 11 mg (F)
**Folic acid (µg)**	502.07 (329.35; 674.80)	397.14 (259.22; 535.05)	400 µg	250 µg dietary folate equivalents
**Vitamin D (µg)**	2.58 (0; 6.64) *	0.80 (0.14; 1.46)	15 µg	5–10 µg (19–65 years)
**Vitamin B6 (mg)**	2.35 (1.95; 2.75)	2.30 (1.67; 2.94)	1.7 mg (M) 1.3 mg (F)	1.3 mg
**Biotin (µg)**	17.06 (13.56; 20.56)	20.50 (14.04; 26.96)	30 µg	30 µg
**Vitamin B12 (µg)**	2.96 (1.13; 4.79)	2.52 (1.14; 3.91)	2.4 µg	2.4 µg
**Vitamin K (µg)**	n.d.	n.d.	140 µg	65 µg (M) 55 µg (F)

Comparing the recommended intake by FBDG with the 95% Confidence Interval of bromatological composition of the 7-DFP, it is evident an upper content of proteins both in Italy and MER. Folic acid, vitamin B6 and Riboflavin reach recommended values in both regions. Under intake in both countries: calcium, vitamin D, biotin, vitamin A. Only in MER sugars, total fats, vitamin E (only for females) slightly exceed recommended values.

EPA and DHA are under suggested values in MER.

## Discussion

This paper, for the first time, compares the prevalence of obesity, overweight and NCD in Italy, Lebanon and Palestine, in relation to adherence to MD and proposes an example of simulated weekly food plan, constructed according to MD criteria, based on local food habits and recipes, which follows the FBDG and takes into account the recommended levels of macro- and micronutrients.

The results show that this type of weekly food plan could be proposed to the population, with the appropriate adjustments for age and gender, both to decrease the risk of developing NCD and as a nutritional medical therapy.

However, it is necessary to pay attention, even when diet plans are designed according to the criteria of the FBDG and the MD, that the maximum recommended protein quota could be very easily exceeded. Excessive protein intake can have various impacts on NCD. High levels of dietary protein, especially from animal sources, can increase inflammatory cytokine levels, which are linked with a higher risk of various NCD. The source of proteins (plant vs. animal) plays a critical role in these outcomes [61]. Excess protein of animal origin is usually associated with excess fat and cholesterol. It is therefore necessary, when proposing a dietary plan according to the criteria described above, to favour protein sources of vegetable origin [62]. This is particularly evident for MER 7-DFP, in which there is a slightly higher amount of total fat than recommended values and, at the same time, lower EPA and DHA contents than recommended values.

Excessive intake of total fats, particularly saturated fatty acids (SFAs), has been consistently linked to an increased risk of various NCD [63].

The type of fat consumed should be considered. Unsaturated fats, found in foods like fish, nuts, and vegetable oils, can decrease the risk of NCD, while trans fats and saturated fats increase risks [64].

Among fats, an important health role is played by the intake of omega-3 FA. Indeed, a meta-analysis conducted by Khan et al. [65] showed a positive association of EPA-DHA, particularly from EPA, as shown in monotherapy-based trials, against cardiovascular outcomes and mortality. The same systematic review suggests that an intake of at least 250 mg per day of omega-3 FA can significantly reduce sudden cardiac death.

In the MEd, EPA and DHA intake was lower than in the Italian diet. These results are in line with the trend reported in the Lebanese guidelines, because fish consumption is lower than the one recommended by WHO guidelines, which recommend a consumption of fatty fish, such as salmon or herring at least once a week, and eventually a plant source of omega-3 FA or supplements [30]. In addition, in areas like Palestine, access to fish may be limited by occupation restrictions [66]. In the presented diet, the two traditional fish recipes, namely “*siyyadiyè*” and “*samkè harra*” involved the use of sea bass, grouper, or mullet, fish not particularly rich in EPA and DHA.

The carbohydrate quality in the diet can also affect health. In fact, a meta-analysis conducted by Huang et al. [67] highlights the association between excessive consumption of sugars, particularly fructose, and adverse cardiometabolic effects, such as increased body weight, and increased ectopic fat. The weekly mean content of sugars in MER 7-DFP is higher than the recommended value by WHO/FAO.

In contrast, fiber intake almost reaches recommendations in both areas. As reported in a meta-analysis by Veronese et al. [68], fibers play a protective function toward CVD. High fiber intake lows glycemic index and exerts a beneficial action on the composition of gut microbiota, which is responsible for the production of short-chain fatty acids (SCFA) [69].

Particularly in the MER, recipes include bread made with refined flour and rice as the main source of grains. Lebanese FBDG reports that, although grains are the basis of the population’s diet, currently, there is a growing trend whereby refined grains, in the form of refined flour, pasta, and rice, are consumed as the main staple. The Lebanese FBDG for increasing fiber intake recommends including at least half a serving per day of whole grains in the diet [30].

Regarding micronutrients, both LARN 2014 and WHO recommend an intake of 1000 mg of calcium per day, which is higher than the average content in the Italian and MER 7-DFP. This result may be dictated by the fact that the calcium intake from water was not taken into account.

The zinc average requirement for MER adults is not reached in the 7-DFP. This micronutrient needs particular attention, since zinc deficiency is still prevalent in the populations [70].

Both the Italian and MER 7-DFP do not reach the recommended 10 µg intake of vitamin D, which could be partly met by endogenous production following sun exposure of the skin [71]. Vitamin D deficiency has been associated with various non-communicable diseases (NCD), as evidenced by recent research [72, 73].

Vitamin D deficiency is an actual issue in Europe, as well as in the MER (Lebanon, Saudi Arabia, Kuwait, Jordan). An epidemiologic study conducted by Rizzoli et al. in Europe, the Middle East, Latin America, the Pacific Coast, and Asia in postmenopausal women with osteoporosis found vitamin D deficiency in 64% of the sample, with a significant association given by ethnicity and phototype, BMI, latitude, supplementation, general health, low health literacy, and generally low education, sun exposure, and lack of recent travel to sunny places. In addition, a study in postmenopausal Lebanese women with osteoporosis compared vitamin D levels between Catholic and Muslim women, which was lower in the latter group. Scholars speculate that the cause could be multifactorial, and in particular that one factor would reside in the traditional dress code that requires covering the arms [74, 75].

The evaluation of real adherence to the MD and its impact on obesity and overweight appears to be of fundamental importance, for the possibility of attributing how different levels of adherence to MD can be associated with and influence the development of NCD, seen in the two different geo cultural contexts. In synthesis, although the association between overweight and obesity with adherence to the MD is, in general, not statistically significant, the estimates suggested that high adherence to MD is a potential protective factor for obesity. Regarding overweight, medium, and low adherence to MD, there is no evidence that it is a risk factor in MER; on the contrary, low adherence seems to be a risk factor in Italy. Nevertheless, some of the studies are characterized by a very wide confidence interval of the OR, showing a low precision of the estimated association between obesity/overweight and adherence to the MD. A greater number of studies with a higher number of subjects with high adherence to the MD should be needed for a better estimation of the association. In particular, few studies referring to MER, avoided obtaining reliable results on the association mentioned above. Moreover, this made it difficult to compare the effect of the low and median adherence to the MD in Italy and MER.

## Conclusion

Our study highlights that it can be possible to follow either Italian or Middle Eastern dietary habits and adhere to MD. High adherence have a significant impact on reducing and controlling NCD, provided that the actual calories consumed do not exceed the total calories requirement.

The aspects relating to caloric needs should also be more emphasized, depending on actual individual energy needs, the exceeding of which prevails over any other parameter that characterizes various dietary models considered healthy, including the MD. In fact, caloric excess strongly influences the increased risk of developing NCD. Moreover, even when a dietary plan is created following the MD criteria and referring to the energy, macro and micronutrient needs, it is still necessary to verify the possible presence of excesses or deficits of nutrients that can have an impact on influencing the risk of developing NCD.

In addition, the results of the present work regarding MD adherence, highlight the need to disseminate the indications for a healthy diet in a widespread and, above all, simple and understandable way for the entire population, regardless of socioeconomic and cultural status.

It is also to be taken into great consideration, how income may affect the real sustainability of the MD for the population from an economic point of view. In fact, in both Italy and MER, the diets have also become increasingly Westernized due to affordability; globalization also conditions the type of cheaper and easily accessible foods, which often lack the characteristics and requirements proposed by the MD.

### Electronic supplementary material

Below is the link to the electronic supplementary material.

Supplementary Material 1

Supplementary Material 2

Supplementary Material 3

Supplementary Material 4

Supplementary Material 5

Supplementary Material 6

## Data Availability

The datasets used and/or analyzed during the current study are available from the corresponding author on reasonable request.

## Abbreviations

BMI: Body mass index
CHD: Coronary heart diseases
CVD: Cardiovascular diseases
FA: Fatty acids
FAO: Food and Agricultural Organization
FBDG: Food-Based Dietary Guidelines
7-DFP: 7-Day Food Plan
MD: Mediterranean diet
MDS: Mediterranean Diet Score
MEd: Middle Eastern diet
MER: Medium East Region
NCD: Non-communicable diseases
WHO: World Health Organization
E%: Total energy percentage
