# Supplementary material for: Italian and Middle Eastern adherence to Mediterranean diet in relation to Body Mass Index and non-communicable diseases: nutritional adequacy of simulated weekly food plans
Source: J Transl Med. 2024 Jul 30;22:703. doi: 10.1186/s12967-024-05325-1 (PMC11290242; doi:10.1186/s12967-024-05325-1)
Supplement: Supplementary file 3 — Supplementary Material 3 [file 12967_2024_5325_MOESM3_ESM.docx]

Supplementary Table 2. The 12 topics covered in the Italian Dietary Guidelines for Healthy Eating

| 1. Control your weight and stay physically active |
| --- |
| 1. More fruits and vegetables |
| 1. More whole grains and legumes |
| 1. Drink plenty of water every day |
| 1. Fats: choose which ones and limit the amount |
| 1. Sugars, sweets, and sugary drinks: less is better |
| 1. Salt: less is better |
| 1. Alcoholic beverages: as little as possible |
| 1. Vary your diet: how and why |
| 1. Special tips |
| 1. Beware of diets and the use of supplements without any scientific basis food safety is also up to you |
| 1. Sustainability of diets: we can all contribute |
