# Supplementary material for: Italian and Middle Eastern adherence to Mediterranean diet in relation to Body Mass Index and non-communicable diseases: nutritional adequacy of simulated weekly food plans
Source: J Transl Med. 2024 Jul 30;22:703. doi: 10.1186/s12967-024-05325-1 (PMC11290242; doi:10.1186/s12967-024-05325-1)
Supplement: Supplementary file 4 — Supplementary Material 4 [file 12967_2024_5325_MOESM4_ESM.docx]

Supplementary Table 3. The 14 key points in the FBDG for Lebanese Adults

| 1. Enjoy and Maintain a Healthy Body Weight |
| --- |
| 1. Be Physically Active Every Day |
| 1. Eat a Variety of Nutritious Foods Every Day for a Balanced Diet |
| 1. Eat Cereals, Especially Whole Grains, as the Basis of Daily Meals |
| 1. Enjoy More Fruit and Vegetables Daily |
| 1. Consume Legume-Based Dishes Regularly and Enjoy Some Unsalted Nuts and Seeds |
| 1. Consume Low-Fat Milk and Dairy Products Every Day |
| 1. Consume at Least Two Servings of Fish, Including Fatty Fish, Every Week |
| 1. Consume Lean Red Meat and Poultry |
| 1. Limit Intake of Sugar, Especially Added Sugar from Sweetened Foods and Beverages |
| 1. Limit Intake of Solid Fats and Replace with Vegetable Oils |
| 1. Limit Intake of Table-Salt and High-Salt Foods |
| 1. Drink Plenty of Safe Water Every Day |
| 1. Eat Safe Food |
