# Supplementary material for: Italian and Middle Eastern adherence to Mediterranean diet in relation to Body Mass Index and non-communicable diseases: nutritional adequacy of simulated weekly food plans
Source: J Transl Med. 2024 Jul 30;22:703. doi: 10.1186/s12967-024-05325-1 (PMC11290242; doi:10.1186/s12967-024-05325-1)
Supplement: Supplementary file 5 — Supplementary Material 5 [file 12967_2024_5325_MOESM5_ESM.docx]

Luconi Table 4 supplementary material. Example of one day nutrition plan for Italian and Lebanese population.

| **ITALY** | **MER** |
| --- | --- |
| **BREAKFAST**  A cup of milk  4 dry cookies  2 teaspoons of sugar | **BREAKFAST**  Pita bread  4 slices of halloum cheese  a cucumber  an orange |
| **SNACK**  One fruit  A package of crackers | **SNACK**  one fruit and one serving of nuts |
| **LUNCH**  A plate of pasta  meat ragout  A teaspoon of parmesan cheese  a portion of mixed salad  2 tablespoons olive oil  bread | **LUNCH**  Rice with a portion of baked fish (sayedie)  mixed salad (fatouch)  two tablespoons of olive oil |
| **SNACK (through the day)**  fruit yogurt | **SNACK (through the day)**  one fruit |
| **DINNER**  Spelt soup with vegetables  One egg  one serving of vegetables  one and a half tablespoons of olive oil  bread  one fruit | **DINNER**  A slice of meat pie (kebbeh)  Salsa with yogurt cucumber and mint  a tablespoon of olive oil  pita bread  fresh fruit  a serving of almonds, peanuts and pistachios. |
