# Supplementary material for: Italian and Middle Eastern adherence to Mediterranean diet in relation to Body Mass Index and non-communicable diseases: nutritional adequacy of simulated weekly food plans
Source: J Transl Med. 2024 Jul 30;22:703. doi: 10.1186/s12967-024-05325-1 (PMC11290242; doi:10.1186/s12967-024-05325-1)
Supplement: Supplementary file 6 — Supplementary Material 6 [file 12967_2024_5325_MOESM6_ESM.pdf]

Università degli Studi di Milano
Dipartimento di Scienze Biomediche per la Salute

Prof.ssa Erna C. Lorenzini
via Mangiagalli 31, 20193 Milano – Italy
erna.lorenzini@unimi.it cell p. +39 3470175151

Dear Editors,

Thank you for the opportunity to submit a revised version of the manuscript **JTRM-D-24-01671**
"Mediterranean Diet: Italian and Middle Eastern Dietary Habits in relation to Non-Communicable Diseases."
Ester Luconi; Martina Tosi; Patrizia Boracchi; Ilaria Colonna; Emilia Rappocciolo; Anita Ferraretto; Erna Cecilia Lorenzini

As regards the revised version of the manuscript, in brief:

- The original text has been modified according to the Reviewers' requests, i.e. by shortening the Introduction and expanding the Discussion
- We clarified some crucial point about how we realized the two dietary plans. We are sorry because we were not clear enough, leading to the interpretation that they result from a survey based on food diaries, while instead they are dietary plans created by dieticians following the criteria of the Mediterranean diet by using meal structures and dishes typical of the Italian and Middle Eastern regions.

We are grateful for the Reviewers' work which has allowed us to improve the quality of our Manuscript.
We hope the Revised version of the Manuscript will fulfill the quality standard for publication on the collection "Planeterranean" of JTRM.

Erna C. Lorenzini

Erna Lorenzini

Milano, 05/05/2024

Erna C. Lorenzini
MD - Clinical Nutrition
PhD Experimental Pathology
Assistant Professor in Applied Dietary Technical Sciences
Università degli Studi di Milano,
Dipartimento di Scienze Biomediche per la Salute
via Luigi Mangiagalli 31 -20133 Milano, Italia
cell phone +39 3470175151
ORCID 0000-0003-4344-4130
