# Supplementary material for: Italian and Middle Eastern adherence to Mediterranean diet in relation to Body Mass Index and non-communicable diseases: nutritional adequacy of simulated weekly food plans
Source: J Transl Med. 2024 Jul 30;22:703. doi: 10.1186/s12967-024-05325-1 (PMC11290242; doi:10.1186/s12967-024-05325-1)
Supplement: Supplementary file 1 — Supplementary Material 1 [file 12967_2024_5325_MOESM1_ESM.docx]

**JTRM-D-24-01671**

**Mediterranean Diet: Italian and Middle Eastern Dietary Habits in relation to Non-Communicable Diseases.**

**Ester Luconi; Martina Tosi; Patrizia Boracchi; Ilaria Colonna; Emilia Rappocciolo; Anita Ferraretto; Erna Cecilia Lorenzini**

**ANSWERS to REVIEWERS’ COMMENTS**

**Reviewer #1**: *I've read with attention the paper of Lorenzini et al. that is potentially of interest. The background and aim of the study have been clearly defined. The methodology applied is overall correct, the results are reliable and adequately discussed. I have no ethical concerns regarding experiments, nor on plagiarism or publication ethics. I've however some concerns:*

*It is not clear the attinence of this kind of research with the aim of the JTM.*

From the home page of the collection "Planeterranean" Diet: Globally Extending the Health Benefits of the Mediterranean Diet”: Globally Extending the Health Benefits of the Mediterranean Diet. Medical evidence supports the pivotal role of the Mediterranean diet in the prevention of non-communicable diseases. Nevertheless, the application of this dietary pattern in non-Mediterranean countries is still challenging. “Planeterranean” is the project of the UNESCO Chair of “Health Education and Sustainable Development”, recently presented with an editorial on the Journal of Translational Medicine, to develop healthier dietary patterns and build up country-specific nutritional pyramids based on locally available foods, presenting the same healthy nutritional properties of Mediterranean Diet.”

One purpose of this work, as reported in the last paragraph of Introduction section of the manuscript revised version, is “implementing two 7-DFP, aligned with MD criteria, utilizing the typical dishes and dietary patterns of the two geographical regions under consideration, it could be possible to identify any deficiencies or excesses of specific nutrients to focus on, in order to reduce the risk of developing diet related NCD” (lines 108-111). We think and hope that these aims could be applied to the special collection. of JTRM. Moreover, we sent the abstract to the Editors’ collection months ago, and we have received a positive answer as it concerns the attinence of our work.

*The abstract should contain some main quantitative data*

We thank the Reviewer for this correct observation. We added some quantitative data in the revised version of our manuscript (lines 29-31)

*The introduction is very long and unfocused. It should be shortened and focused helping the reader to immediately understand why a comparison between Italian and Palestinian diets are compared*.

We thank the Reviewer and agree with this suggestion. In the revised version of the manuscript a great effort has been made to short, make more comprehensive and focused the Introduction, even by eliminating some paragraphs and adding new ones.

*Table 1: where data obtained in different countries compared in some way? If yes, how? If no, it should be done*

In the revised version Table 3 (previous Table 1) according to the test used to evaluate the differences, the following information is reported: the mean and the standard deviation of the amount of nutrients in Italy and Middle Eastern countries, the mean difference between Italy and Middle Eastern countries (with its 95% Confidence Interval), and the p-value is reported when the t-test is used.

When the Wilcoxon’s test was used, the table contains the median (first and third quartile) of the amount of the nutrient in Italy and in Middle Eastern countries, the difference in location (Italy-Middle East) with its 95% Confidence Interval, and the p-value"

*The meta-analysis is deeply described in the text and with a number of data, but often it is based on 2 studies only, and this limit the interest of the observation. I think that this part should be also dramatically shortened.*

Thank you for your comment. We have shortened the paper with the remotion of the parts about physical activity, cholesterol, and triglycerides, as the studies that met the inclusion criteria were only about Italy or the Middle East and were few. Therefore, comparing the association between those items and the levels of adherence to the Mediterranean Diet between the two countries would not have been possible.

*Overall the authors should make more effort to harmonize the paper because in the current version it seems to be made of 2 short communication and a full paper.*

We agree with the Reviewer, a special effort has been made in the revised version of the manuscript to harmonize all the parts.

**Reviewer #2**: *The authors conducted a study on Mediterranean Diet and Non-Communicable Diseases, comparing Italian and Middle Eastern population. The issues addressed, however, are not novel, as the role of the Mediterranean diet in non-communicable diseases and comparisons among populations bordering the Mediterranean Sea have already been extensively investigated.*

We were conscious that the issue addressed is not novel, but we aimed to try to explain the paradox among Mediterranean diet adopted by food based dietary guidelines, which contras with real food intake, and high prevalence of obesity, overweight and non communicable diseases in both areas. We also compared two possible seven-day food plans, following Mediterranean diet pattern, by using meal structures and local dishes (Italian or Middle Eastern), to identify any deficiencies or excesses of macro and micronutrients that can affect non-communicable diseases.

*The background is comprehensive about Mediterranean Diet, but not sufficient on Non-Communicable Disease. They are in fact scarcely mentioned,* *the impact they have on people's health is not highlighted in any way, and* *the factors that lead to these diseases are not mentioned. It is therefore unclear why the authors were prompted to conduct this study*.

Thank you for these considerations. As suggested, we have modified the introduction, inserting “*the factors that lead to these diseases”* (lines 44-46); “*the impact they have on people's health is not highlighted* “ (lines 76-89).

*Moreover, although the interesting authors aim "to compare: the macro- and micronutrient composition of the Italian diet with that of the typical Middle Eastern one, in particular Lebanon and Palestine, based on the criteria of the Mediterranean diet; the impact of different levels of adherence to the Mediterranean diet, in the two different areas, on some anthropometric and hematochemical parameters markers of non-communicable diseases; the Italian guidelines with those of Lebanon.", the study design is not at all clear and the materials and methods are confusing*.

We thank the Reviewer, indeed the observations are absolutely right. We completely rewrote the materials and methods part, also including two flow charts to clarify the study design (Figure 1 and 2). We hope the new version could be clearer-

*Indeed, the source of the data is not specified; it is not clear whether data from projects were used, or whether an actual study was conducted, or whether a survey was conducted. It is not clear who compiled the 7-day food diary (7-DFD).*

Thank you for your request, we are sorry because we were not clear at all on how we realized the two 7-DFD. We would like to clarify that now renamed 7-day food plan (7-DFP), are not a result of a survey based on self-reported food diaries, compiled by a population sample, but are actually two dietary plans developed by Italian dietitians and dietitians from Birzeit University, following the specific nutrition guidelines for Mediterranean diet in the two different countries. Please, see revised section Material and Methods (lines 201-220).

*The Italian guidelines cited are not those specified by the Italian Ministry of Health*.

In our work we referred to “Linee guida per una sana alimentazione italiana - 2018” (ISBN 9788833850375) cited by Italian Ministry of Health as a reference guidelines, which can be accessed via the following link: https://www.salute.gov.it/portale/documentazione/p6_2_2_1.jsp?id=2915

**Reviewer #4: Dear author,**

*I appreciate the opportunity to review the manuscript titled "Mediterranean Diet: Italian and Middle Eastern Dietary Habits in relation to Non-Communicable Diseases." Substantial improvements are required before the work can be deemed suitable for potential publication. Enclosed are my observations and recommendations aimed at enhancing the clarity and quality of the manuscript. Please find below my insights for your consideration.*

*OVERALL LENGTH AND READABILITY:*

*The manuscript appears too long, which distracts the reader from its main findings and messages. Simplifying the content and improving readability through condensing sections, removing redundancies, and prioritizing key points could enhance reader engagement. Additionally, the study design should be clarified throughout the manuscript. The current presentation lacks explicit details about the study design, which may hinder readers' understanding of the research methodology. Furthermore, revising the title to accurately reflect the study's content and scope is recommended, considering the need for clarification on the study design*.

Thank you very much for all suggestions. We have tried to make our work clearer.

“We have changed the title as: “Italian and Middle Eastern adherence to Mediterranean diet in relation to Body Mass Index and Non Communicable Diseases: nutritional adequacy of simulated weekly food plans.”

We rewrote and condensed all parts of the manuscript, including flow charts with the study design (Figures 1 and 2)

BACKGROUND:

*Considering the extensive background in your manuscript, you might simplify this section to focus more directly on aspects of the Mediterranean diet relevant to noncommunicable diseases, particularly through a concise comparison of Italian and Middle Eastern dietary habits*.

We have reduced the background and focused on those aspects of the Mediterranean Diet most related to non-communicable diseases (lines 87-93) and the main pathogenetic mechanisms link to non communicable diseases (lines 44-46).

*-For instance, emphasizing dietary fat intake differences or the role of plant-based foods might directly tie to your objectives*

In the revised manuscript, we reported fat intakes as percentages of total energy in the Italian and Middle Eastern population, as taken from the literature (lines 253-263). We also compared the bromatological composition of the two diet plans we implemented (7-DFP) and highlighted the differences, among them the fat content, including the possible inadequate presence of EPA and DHA (Table 3, and lines 414; 417; 456-458).

METHODS:

*Consolidate all references to the use of R software exclusively within the 'Statistical analysis' subsection, specifying the statistical models and tests applied, to streamline the methodology narrative.*

Thank you for your comment. We have meticulously consolidated all references to the use of R within the “Statistical analysis” section. Our revisions have not only made the section clearer, but also provided a more comprehensive description of the meta analysis. We have also included the description of the forest plot, the comparison of the micro and macronutrients of the two dietary models, and the comparison with the guidelines of the quantity of macro and micronutrients of the two diet models.

*Clarify the selection criteria for the dietary guidelines and recipes, particularly the choice of Lebanese and Palestinian recipes.*

Thank you for your requests, we are sorry because we were not clear at all on how we realized the two 7-DFD. We would like to clarify that now renamed 7-day food plan (7-DFP), are not a result of a survey based on self-reported food diaries, compiled by a population sample, but are actually two dietary plans developed by Italian dietitians and dietitians from Birzeit University, following the specific nutrition guidelines for Mediterranean diet in the two different countries. Please, see revised section Material and Methods (lines 201-220).

*and justify the selection of MetadietaVR software for nutritional analysis, ensuring readers understand the rationale behind these choices*.

We explained the choice of Metadieta software (lines 211-202): “The two 7-DFP were analyzed using a software (MetadietaVR; METEDAsrl, via S. Pellico 4, San Benedetto del Tronto, AP, Italy). Metadieta software includes a vast database of food items with portion photos, constantly updated and implemented with values from the main official databases as: From Italy, Council for Agricultural Research and Agricultural Economics [34]; Also from Italy, BDA-IEO [35]; U.S. Deparment of Agriculture [36]; CIQUAL French Food Composition [37]. It is commonly used in the dietetic clinical practice inside hospitals and during outpatient activities, by dietitians and nutritionists. In the present work, it was specifically chosen MD adherent food items, respecting dietary habits and ensuring that the nutritional composition of meals reflected the usual pattern and recipes.”

*Reorganize the subsection on 'Study selection and eligibility criteria' to directly follow the introduction of the literature search, creating a logical progression from search strategy to study inclusion. Eliminate any repetitive details across the subsections, especially regarding the software and analysis methods, to avoid redundancy and improve readability*.

Thank you for your comment. We have rewritten the statistical analysis methods to improve readability and avoid redundancies

RESULTS:

*The Results section, including the figures, appears confusing due to the presence of some missing figures, others embedded within the text, and additional ones located at the end of the manuscript. Furthermore, there are sentences that appear to be unrelated to the main findings. Please review this section to eliminate confusion, rectify any errors, and ensure that all content is relevant to the study outcomes.*

In the revised manuscript, some results were removed because, as requested by other reviewers, the data between the two geographic areas were insufficient for comparison. See flow chart in Figure 2. All Figures and Tables are no more embedded within the text (uploaded as separate files).

*You mentioned that "high adherence to MD is associated with high mean levels of total, LDL, and HDL cholesterol in both populations," but it's unclear where these results are presented. Please provide statistically valid evidence to support these claims.*

Thank you for your comment. We have removed the parts about the physical activity, cholesterol and triglycerides, as the studies that met the inclusion criteria were only about Italy or the Middle East and therefore a comparison of the association between those items and the levels of adherence to the Mediterranean Diet between the two countries would not have been possible.

*Consider relocating the detailed comparison between the* *Italian and Lebanese dietary guidelines to the supplementary materials. Additionally, contemplate transferring less relevant results to the supplementary material.*

We agree with the Reviewer suggestions and both Italian and Lebanese dietary guidelines have been transferred to the supplementary material (Supplementary Tables 2 and 3)

*Implementing these adjustments would enable the main results section to concentrate more directly on the primary findings related to nutrient composition and health outcomes.*

*DISCUSSION: To enhance the impact of your 'Discussion' section, please directly address the implications of the significant dietary differences, particularly the lower omega-3 fatty acid intake in the Middle Eastern diet versus the Italian diet, and its potential public health impacts.*

Thank you, we have tried to explain our data and some public implications (lines 461-474): “The type of fat consumed should be considered. Unsaturated fats, found in foods like fish, nuts, and vegetable oils, can decrease the risk of NCD, while trans fats and saturated fats increase risks [64].

Among fats, an important health role is played by the intake of omega-3 FA. Indeed, a meta-analysis conducted by Khan et al. [65] showed a positive association of EPA-DHA, particularly from EPA, as shown in monotherapy-based trials, against cardiovascular outcomes and mortality. The same systematic review suggests that an intake of at least 250 mg per day of omega-3 FA can significantly reduce sudden cardiac death.

In the MEd, EPA and DHA intake was lower than in the Italian diet. These results are in line with the trend reported in the Lebanese guidelines, because fish consumption is lower than the one recommended by WHO guidelines, which recommend a consumption of fatty fish, such as salmon or herring at least once a week, and eventually a plant source of omega-3 FA or supplements [30]. In addition, in areas like Palestine, access to fish may be limited by occupation restrictions [66]. In the presented diet, the two traditional fish recipes, namely "siyyadiyè" and "samkè harra" involved the use of sea bass, grouper, or mullet, fish not particularly rich in EPA and DHA*.”*

*Furthermore, a more focused examination of the unexpected findings, such as the higher levels of total and LDL cholesterol despite high adherence to the Mediterranean diet, is crucial (if this is true)*

As we mentioned above, we have removed the parts about the physical activity, cholesterol (LDL included) and triglycerides, as the studies that met the inclusion criteria were only about Italy or the Middle East and therefore a comparison of the association between those items and the levels of adherence to the Mediterranean Diet between the two countries would not have been possible.

*Please identify and condense or eliminate any repetitive sections within the 'Discussion' that reiterate results without offering new analysis or implications. Focusing on novel insights and implications for future research or practice will enhance the section's clarity and impact.*

*To further enhance your 'Discussion' section, I recommend explicitly detailing the strengths of your study, such as its innovative comparative approach and methodological rigor. Additionally, addressing potential limitations, like the reliance on self-reported dietary data that might introduce recall bias, will provide a comprehensive perspective.*

In this work we compared the energy and nutrient content of a simulated weekly diet plan using Italian and Middle Eastern references in terms of dishes and recipes, which observed the principles of the Mediterranean diet, but we pointed out that, even when a dietary plan is created following the MD criteria and referring to the energy, macro and micronutrient needs, it is still necessary to verify the possible presence of excesses or deficits of nutrients that can have an impact on influencing the risk of developing NCD. (lines 530-533). One of the limitations of this work is that it would require an actual diet plan, not the result of a survey in a population sample, which is a limitation.

All comparisons were made following a rigorous statistical methodology, both in the parts relating to meta-analysis, degrees of adherence to the Mediterranean diet and BMI, and in the comparison of food plans, as reported in detail in the text (see revised version of Materials and Methods)

**Reviewer #5**: *The manuscript by Luconi and colleagues focused on the comparison between health and dietary habits of Italian and Middle Eastern populations. In particular, the authors aimed to highlight the difference in the adherence to Mediterranean Diet (MD).*

*General comments*

*The topic is interesting but not novel. Indeed, there is huge evidence in different locations (including countries bordering Eastern Mediterranean Sea). Therefore, the authors should add information about the added value of their findings.*

We would like to thank the Reviewer which gave us the possibility to clarify a new perspective of our work. In fact, we compared the energy and nutrient content of a simulated weekly diet plan using Italian and Middle Eastern references in terms of dishes and recipes, which observed the principles of the Mediterranean diet, but we pointed out that, even when a dietary plan is created following the MD criteria and referring to the energy, macro and micronutrient needs, it is still necessary to verify the possible presence of excesses or deficits of nutrients that can have an impact on influencing the risk of developing NCD. (lines 530-533). One of the limitations of this work is that it would require an actual diet plan, not the result of a survey in a population sample, which is a limitation.

All comparisons were made following a rigorous statistical methodology, both in the parts relating to meta-analysis, degrees of adherence to the Mediterranean diet and BMI, and in the comparison of food plans, as reported in detail in the text (see revised version of Materials and Methods)

*Specific comments*

*Introduction. The section is too long and does not really highlight the relationship between adherence to MD and Non-Communicable Diseases*

We thank the Reviewer and agree with these suggestions. In the revised version of the manuscript a great effort has been made to short, make more comprehensive and focused the Introduction, even by eliminating some paragraphs and adding new ones. As suggested, we have modified the introduction, inserting “the factors that lead to these diseases” (lines 44-46); the impact they have on people's health (lines 76-89). We also pointed out “adherence to the MD has also been shown to be associated with a lower prevalence of metabolic syndrome [18] and lower incidence and mortality from CVD, as published in the "Seven Countries Study”. This study highlights how mortality due to coronary heart disease (CHD) was 2-3 times lower in Mediterranean countries.” (lines 90-94)

*The study design is not completely clear, and methods need further clarification (i.e., who completed the 7-day food diary, number of subjects and drop-out, if any).*

*The authors missed some important information about the study population (i.e., age, gender, anthropometric parameters).*

*Thank you for your request, we are sorry because we were not clear at all on how we realized the two 7-DFD. We would like to clarify that now renamed 7-day food plan (7-DFP), are not a result of a survey based on self-reported food diaries, compiled by a population sample, but are actually two dietary plans developed by Italian dietitians and dietitians from Birzeit University, following the specific nutrition guidelines for Mediterranean diet in the two different countries. Please, see revised section Material and Methods (lines 201-220).*

*Did the authors assess physical activity in the study population? If yes, how? If no, please add it as a limitation of this study.*

Thank you for your comments. Although we are conscious about the importance of physical activity and its impact on obesity and overweight, and consequently, no communicable diseases (lines 508), our study do not derived from a survey but from meta analysis, apart from the realization of the 7 days food plans. We have removed the parts about the physical activity as well as, cholesterol and triglycerides, as the studies that met the inclusion criteria were only about Italy or the Middle East and therefore a comparison between the two countries would not have been possible*.*

*Figures. Some figures are missing/wrong (i.e., figure 11) and many need further description in the text****.***

All tables and figures have been modified and newly attached to the revised manuscript.

*Discussion. The section contains redundant information. Please the authors to highlight novel insights and implications for future research or clinical practice*.

In revising the manuscript we have modified the discussion as suggested by the reviewers' comments, in order to make it clearer. (lines 530-533) (538-540).
